# Deep image reconstruction from human brain activity

**DOI:** 10.1371/journal.pcbi.1006633

**Published:** 2019-01-14

**Authors:** Guohua Shen, Tomoyasu Horikawa, Kei Majima, Yukiyasu Kamitani

**Affiliations:** 1 Computational Neuroscience Laboratories, Advanced Telecommunications Research Institute International, Kyoto, Japan; 2 Graduate school of Informatics, Kyoto University, Kyoto, Japan; Oxford University, UNITED KINGDOM

## Abstract

The mental contents of perception and imagery are thought to be encoded in hierarchical representations in the brain, but previous attempts to visualize perceptual contents have failed to capitalize on multiple levels of the hierarchy, leaving it challenging to reconstruct internal imagery. Recent work showed that visual cortical activity measured by functional magnetic resonance imaging (fMRI) can be decoded (translated) into the hierarchical features of a pre-trained deep neural network (DNN) for the same input image, providing a way to make use of the information from hierarchical visual features. Here, we present a novel image reconstruction method, in which the pixel values of an image are optimized to make its DNN features similar to those decoded from human brain activity at multiple layers. We found that our method was able to reliably produce reconstructions that resembled the viewed natural images. A natural image prior introduced by a deep generator neural network effectively rendered semantically meaningful details to the reconstructions. Human judgment of the reconstructions supported the effectiveness of combining multiple DNN layers to enhance the visual quality of generated images. While our model was solely trained with natural images, it successfully generalized to artificial shapes, indicating that our model was not simply matching to exemplars. The same analysis applied to mental imagery demonstrated rudimentary reconstructions of the subjective content. Our results suggest that our method can effectively combine hierarchical neural representations to reconstruct perceptual and subjective images, providing a new window into the internal contents of the brain.

## Author summary

Machine learning-based analysis of human functional magnetic resonance imaging (fMRI) patterns has enabled the visualization of perceptual content. However, prior work visualizing perceptual contents from brain activity has failed to combine visual information of multiple hierarchical levels. Here, we present a method for visual image reconstruction from the brain that can reveal both seen and imagined contents by capitalizing on multiple levels of visual cortical representations. We decoded brain activity into hierarchical visual features of a deep neural network (DNN), and optimized an image to make its DNN features similar to the decoded features. Our method successfully produced perceptually similar images to viewed natural images and artificial images (colored shapes and letters), whereas the decoder was trained only on an independent set of natural images. It also generalized to the reconstruction of mental imagery of remembered images. Our approach allows for studying subjective contents represented in hierarchical neural representations by objectifying them into images.

## Introduction

While the externalization of states of the mind is a long-standing theme in science fiction, it is only recently that the advent of machine learning-based analysis of functional magnetic resonance imaging (fMRI) data has expanded its potential in the real world. Although sophisticated decoding and encoding models have been developed to render human brain activity into images or movies, the methods are essentially limited to image reconstructions with low-level image bases [1, 2], or to matching to exemplar images or movies [3, 4], failing to combine the visual features of multiple hierarchical levels. While several recent approaches have introduced deep neural networks (DNNs) for the image reconstruction task, they have failed to fully utilize hierarchical information to reconstruct visual images [5, 6]. Furthermore, whereas categorical decoding of imagery contents has been demonstrated [7, 8], the reconstruction of internally generated images has been challenging.

The recent success of DNNs provides technical innovations to study the hierarchical visual processing in computational neuroscience [9]. Our recent study used DNN visual features as a proxy for the hierarchical neural representations of the human visual system and found that a brain activity pattern measured by fMRI could be decoded (translated) into the response patterns of DNN units in multiple layers representing the hierarchical visual features given the same input [10]. This finding revealed a homology between the hierarchical representations of the brain and the DNN, providing a new opportunity to utilize the information from hierarchical visual features.

**Fig 1:**
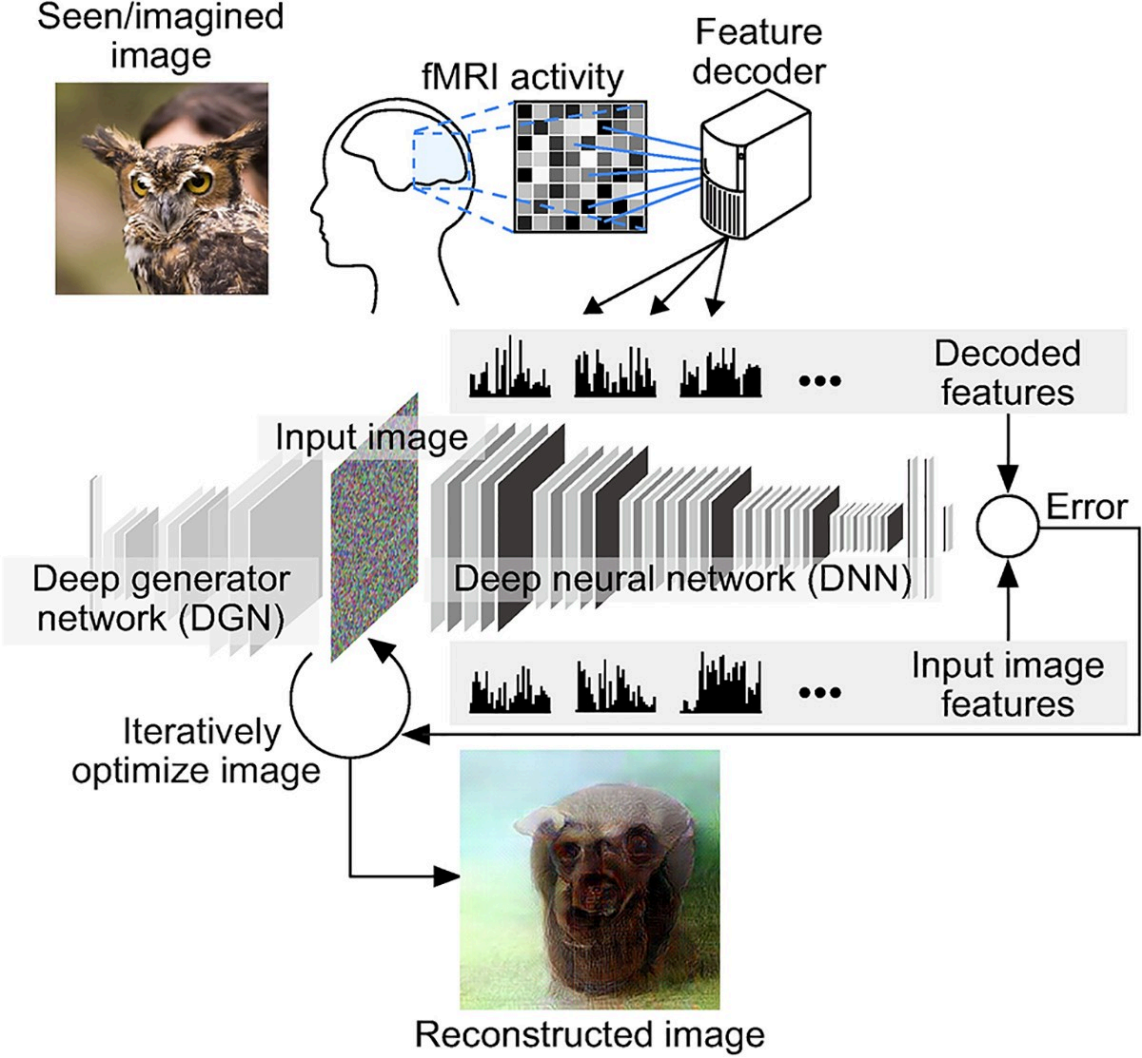
Deep image reconstruction. An overview of a deep image reconstruction is shown. The pixel values of the input image are optimized so that the DNN features of the image are similar to those decoded from fMRI activity. A deep generator network (DGN) is optionally combined with the DNN to produce natural-looking images, in which optimization is performed at the input space of the DGN.

Here, we present a novel approach, named deep image reconstruction, to visualize perceptual content from human brain activity. This technique combines the DNN feature decoding from fMRI signals with recently developed methods for image generation from the machine learning field (Fig 1) [11]. The reconstruction algorithm starts with a given initial image and iteratively optimizes the pixel values so that the DNN features of the current image become similar to those decoded from brain activity across multiple DNN layers. The resulting optimized image is considered as a reconstruction from the brain activity. We optionally introduced a deep generator network (DGN) [12] to constrain the reconstructed images to look similar to natural images by performing optimization in the input space of the DGN.

## Results

We trained the decoders that predicted the DNN features of viewed images from fMRI activity patterns following the procedures of Horikawa & Kamitani (2017) [10]. In the present study, we used the *VGG19* DNN model [13], which consisted of sixteen convolutional layers and three fully connected layers and was pre-trained with images in *ImageNet* [14] to classify images into 1,000 object categories (see Materials and Methods: “Deep neural network features” for details). We constructed one decoder for a single DNN unit to predict outputs of the unit. We trained decoders corresponding to all the units in all the layers (see Materials and Methods: “DNN feature decoding analysis” for details).

The feature decoding analysis was performed with fMRI activity patterns in visual cortex (VC) measured while subjects viewed or imagined visual images. Our experiments consisted of the training sessions in which only natural images were presented and the test sessions in which independent sets of natural images, artificial shapes, and alphabetical letters were presented. In another test session, a mental imagery task was performed. The decoders were trained using the fMRI data from the training sessions, and the trained decoders were then used to predict DNN feature values from the fMRI data of the test sessions (the accuracies are shown in S1 Fig).

Decoded features were then forwarded to the reconstruction algorithm to generate an image using variants of gradient descent optimization (see Material and Methods: “Reconstruction from a single DNN layer” and “Reconstruction from multiple DNN layers” for details). The optimization was performed to minimize the error between multi-layer DNN features decoded from brain activity patterns and those calculated from the input image by iteratively modifying the input image. For natural image reconstructions, to improve the “naturalness” of reconstructed images, we further introduced the constraint using a deep generator network (DGN) derived from the generative adversarial network algorithm (GAN) [15], which is known to capture a latent space explaining natural images [16] (see Material and Methods: “Natural image prior” for details).

**Fig 2:**
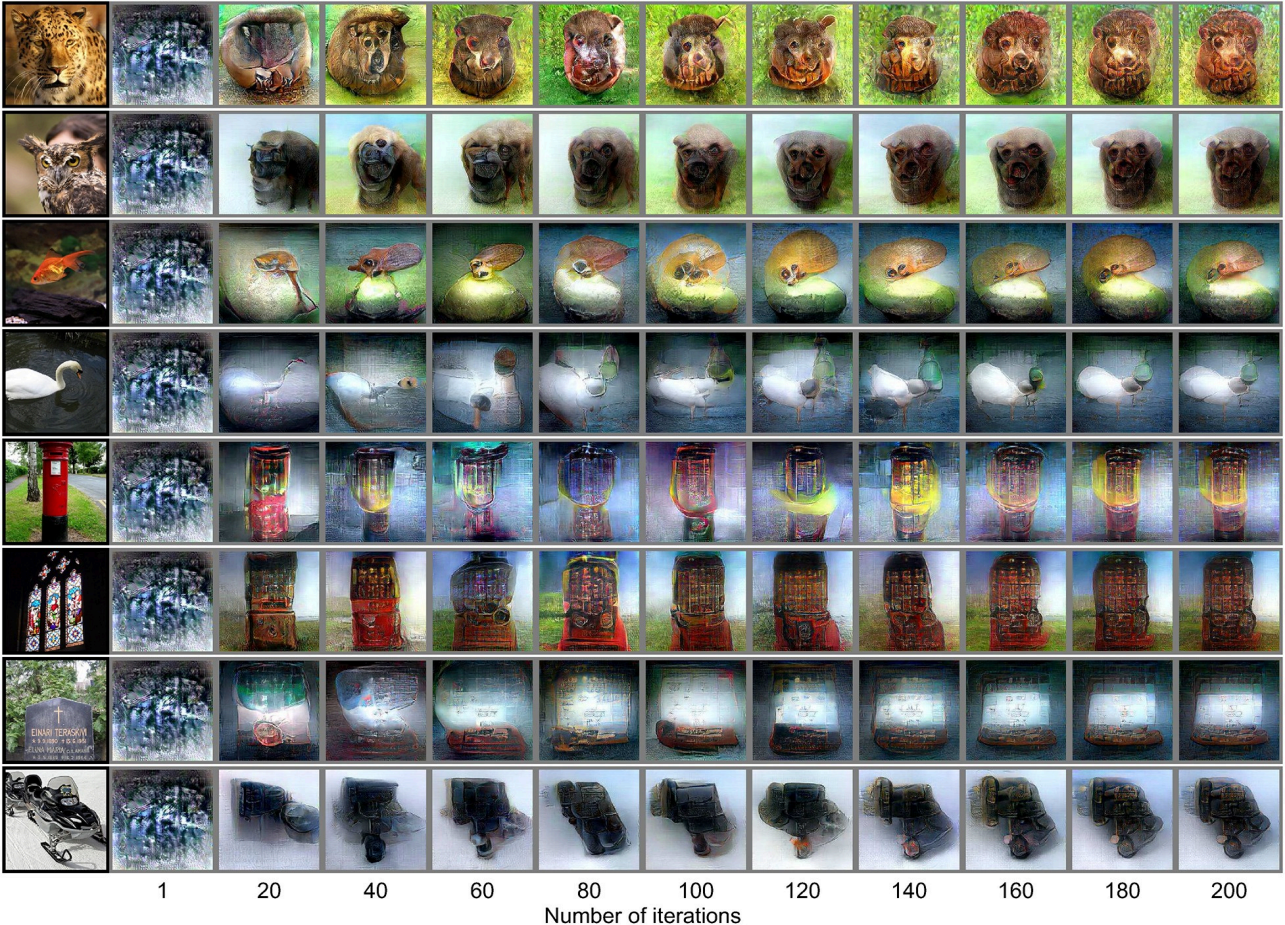
Seen natural image reconstructions. The black and gray surrounding frames indicate presented and reconstructed images respectively (reconstructed from VC activity using DNN1–8). Reconstructed images obtained through the optimization processes are shown for seen natural images. Reconstructions were constrained by the DGN.

Examples of reconstructions for natural images are shown in Fig 2 (see S2 Fig for more examples, and see S1 Movie for reconstructions through the optimization processes). The reconstructions obtained with the DGN capture the dominant structures of the objects within the images. Furthermore, fine structures reflecting semantic aspects like faces, eyes, and texture patterns were also generated in several images. Our extensive analysis on each of the individual subjects demonstrated replicable results across the subjects. Moreover, the same analysis on a previously published dataset [10] also replicated qualitatively similar reconstructions to those in the present study (S3 Fig).

**Fig 3:**
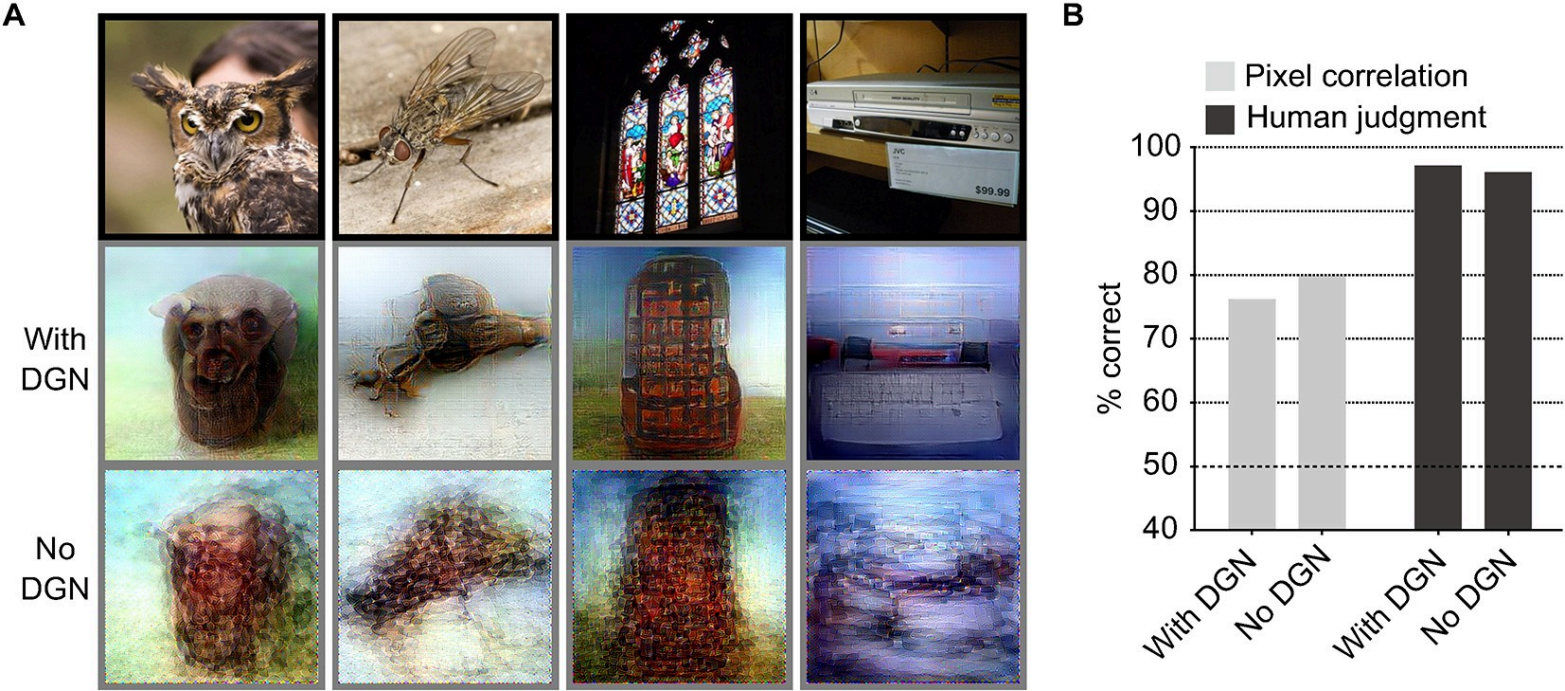
Effect of the deep generator network (DGN). (**A**) Reconstructions with and without the DGN. The first, second, and third rows show presented images, and reconstructions with and without the DGN respectively (reconstructed from VC activity, DNN1–8). (**B**) Reconstruction quality of seen natural images (three subjects pooled, *N* = 150; chance level, 50%).

To investigate the effect of the DGN, we evaluated the quality of reconstructions generated both with and without using it (Fig 3A and 3B; see S4 Fig for individual subjects; see Material and Methods: “Evaluation of reconstruction quality”). While the reconstructions obtained without the DGN also successfully reproduced rough silhouettes of dominant objects, they did not show semantically meaningful appearances (see S5 Fig for more examples; also see S6 Fig for reconstructions from different initial states for both with and without the DGN). Evaluations using pixel-wise spatial correlation and human judgment both showed almost comparable accuracy for reconstructions with and without the DGN (accuracy of pixel-wise spatial correlation, with and without the DGN, 76.1% and 79.7%; accuracy of human judgment, with and without the DGN, 97.0% and 96.0%). However, reconstruction accuracy evaluated using pixel-wise spatial correlation showed slightly higher accuracy with reconstructions performed without the DGN than with the DGN (two-sided signed-rank test, *P* < 0.01), whereas the opposite was observed for evaluations by human judgment (two-sided signed-rank test, *P* < 0.01). These results suggest the utility of the DGN that enhances the perceptual similarity of reconstructed images to target images by rendering semantically meaningful details in the reconstructions.

**Fig 4:**
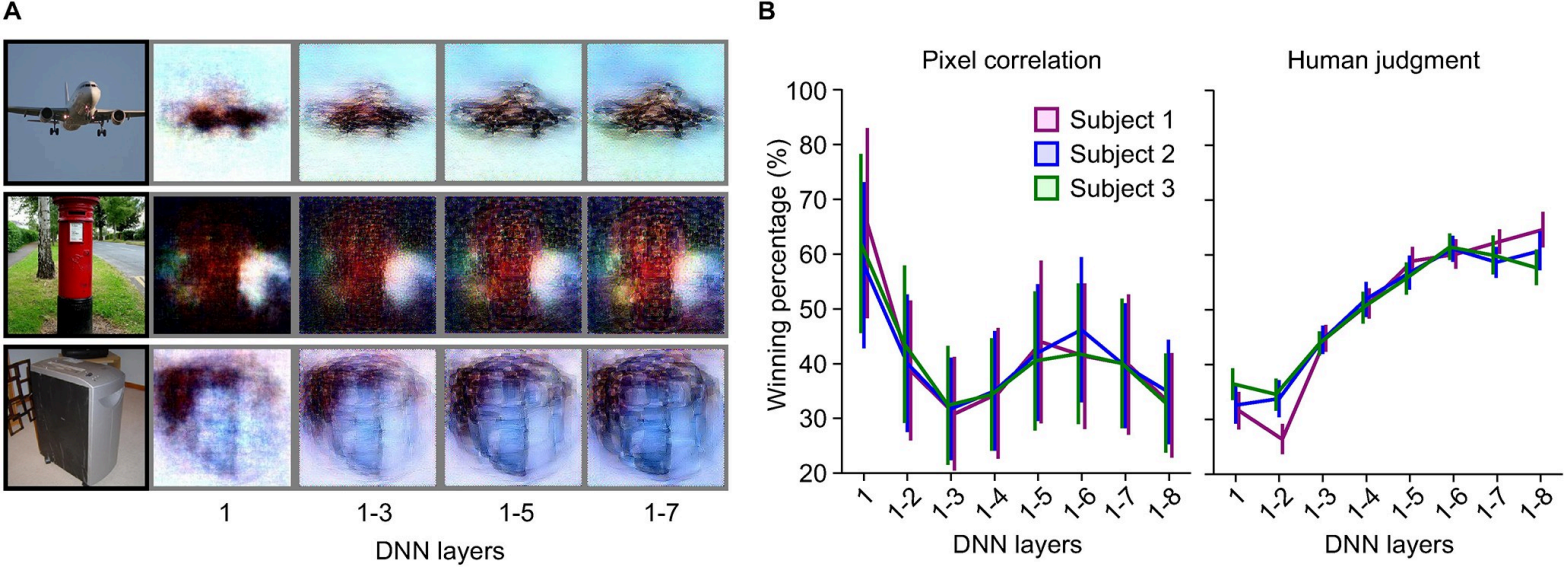
Effect of multi-level visual features. (**A**) Reconstructions using different combinations of DNN layers (without the DGN). The black and gray surrounding frames indicate presented and reconstructed images respectively (reconstructed from VC activity). (**B**) Objective and subjective assessments of reconstructions from different combinations of DNN layers (error bars, 95% confidence interval [C.I.] across samples, *N* = 50; see Material and Methods: “Evaluation of reconstruction quality” for the procedure to calculate winning percentage).

To characterize the ‘deep’ nature of our method, the effectiveness of combining multiple DNN layers was tested using both objective and subjective assessments [5, 17, 18]. For each of the 50 test natural images, reconstructed images were generated with a variable number of multiple layers (Fig 4A; DNN1 only, DNN1–2, DNN1–3, …, DNN1–8; see S7 Fig for more examples). In the objective assessment, the pixel-wise spatial correlations to the original image were compared between two combinations of DNN layers. In the subjective assessment, an independent rater was presented with an original image and a pair of reconstructed images, both from the same original image but generated with different combinations of multiple layers, and was required to indicate which of the reconstructed images looked more similar to the original image. While the objective assessment showed higher winning percentages for the earliest layer (DNN1) alone, the subjective assessment showed increasing winning percentages for a larger number of DNN layers (Fig 4B). Our additional analysis showed poor reconstruction quality from individual layers especially from higher layers (see S8 Fig for reconstructions from individual layers). These results suggest that combining multiple levels of visual features enhanced the perceptual reconstruction quality even though the pixel-wise accuracy is lost.

Given the true DNN features, instead of decoded features, as the input, the reconstruction algorithm produces almost complete reconstructions of original images (S8 Fig), indicating that the DNN feature decoding accuracy would determine the quality of reconstructed images. To further confirm this, we calculated the correlation between the feature decoding accuracy and the reconstruction quality for individual images (S9 Fig). The analyses showed positive correlations for both the objective and subjective assessments, suggesting that improving feature decoding accuracy could improve reconstruction quality.

**Fig 5:**
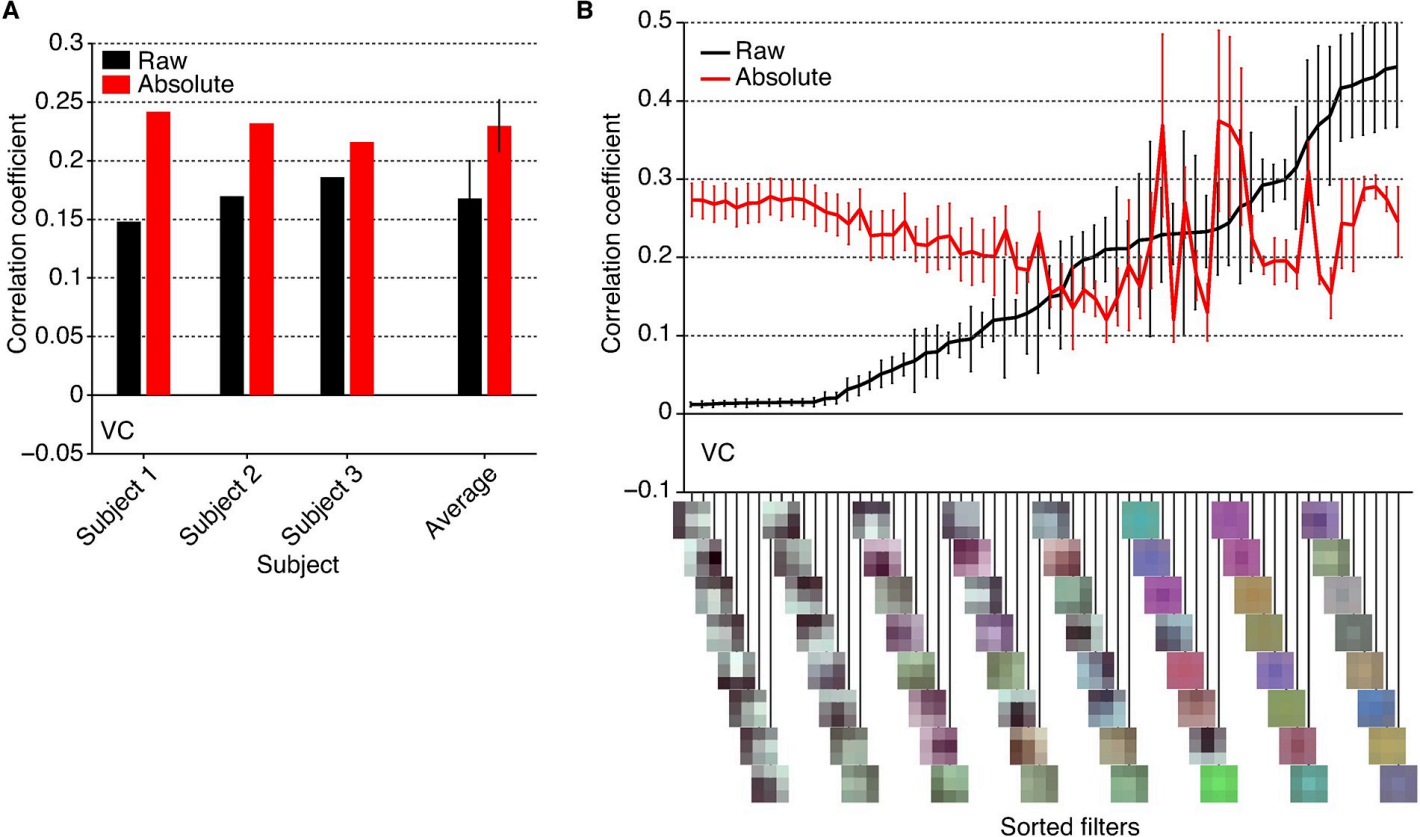
DNN feature decoding accuracy of raw and absolute features. The analysis was performed with features from the conv1_1 layer of the VGG19 model using the test natural image dataset (error bar, 95% C.I. across subjects). (**A**) Mean feature decoding accuracy of all units. (**B**) Mean feature decoding accuracy for individual filters. The feature decoding accuracies of units within the same filters were individually averaged. The filters were sorted according to the ascending order of the raw feature decoding accuracy averaged for individual filters.

We found that the luminance contrast of a reconstruction was often reversed (e.g., the stained-glass images in Fig 2), presumably because of the lack of (absolute) luminance information in the fMRI signals, even in the early visual areas [19]. Additional analyses revealed that the feature values of filters with high luminance contrast in the earliest DNN layers (conv1_1 in VGG19) were better decoded when they were converted to absolute values (Fig 5A and 5B), demonstrating a clear discrepancy between the fMRI and raw DNN signals. The large improvement levels demonstrate the insensitivity of fMRI signals to pixel luminance, suggesting the linear-nonlinear discrepancy of DNN and fMRI responses to pixel luminance. This discrepancy may explain the reversal of luminance observed in several reconstructed images. While this may limit the potential for reconstructions from fMRI signals, the ambiguity might be resolved by modelling DNNs to fill the gaps between signals of DNNs and fMRI. Alternatively, further emphasis of the high-level visual information in hierarchical visual features may help to resolve the ambiguity of luminance by incorporating information on semantic context.

**Fig 6:**
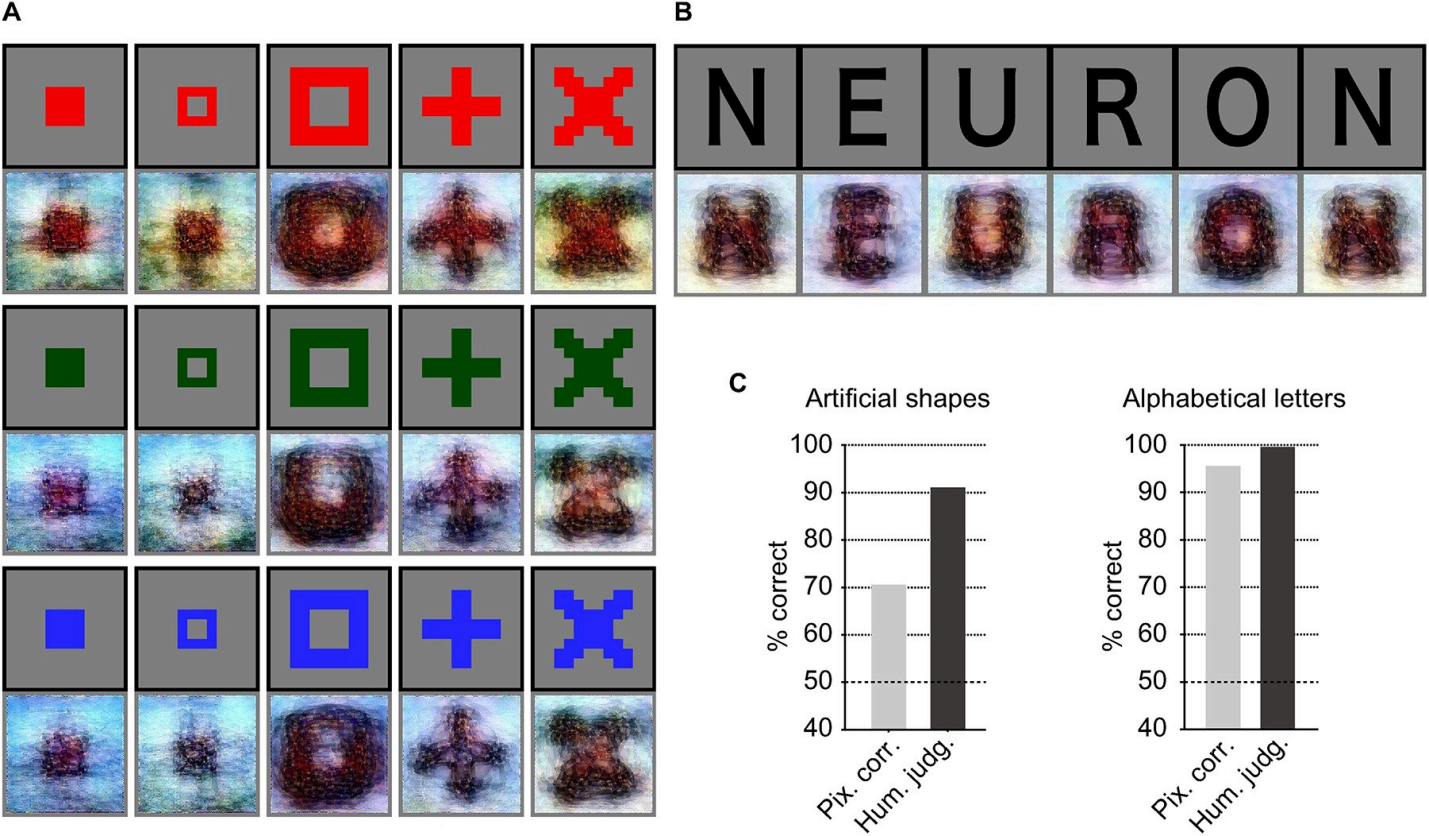
Seen artificial image reconstructions. The black and gray surrounding frames indicate presented and reconstructed images respectively (VC activity, DNN 1–8, without the DGN). (**A**) Reconstructions for seen artificial shapes. (**B**) Reconstructions for seen alphabetical letters. The reconstructed letters were arranged in the word: “NEURON”. (**C**) Reconstruction quality of artificial shapes and alphabetical letters (three subjects pooled, *N* = 120 and 30 for artificial shapes and alphabetical letters, respectively; chance level, 50%).

To confirm that our method was not restricted to the specific image domain used for the model training, we tested whether it was possible to generalize the reconstruction to artificial images. This was challenging, because both the DNN and our decoding models were solely trained on natural images. The reconstructions of artificial shapes and alphabetical letters are shown in Fig 6A and 6B (also see S10 Fig and S2 Movie for more examples of artificial shapes, and see S11 Fig for more examples of alphabetical letters). The results show that artificial shapes were successfully reconstructed with moderate accuracy (Fig 6C left; 70.5% by pixel-wise spatial correlation, 91.0% by human judgment; see S12 Fig for individual subjects) and alphabetical letters were also reconstructed with high accuracy (Fig 6C right; 95.6% by pixel-wise spatial correlation, 99.6% by human judgment; see S13 Fig for individual subjects). These results indicate that our model did indeed ‘reconstruct’ or ‘generate’ images from brain activity, and that it was not simply making matches to exemplars. Furthermore, the successful reconstructions of alphabetical letters demonstrate that our method can expand the possible states of visualizations, with advance in resolution over reconstructions performed in previous studies [1, 20].

**Fig 7:**
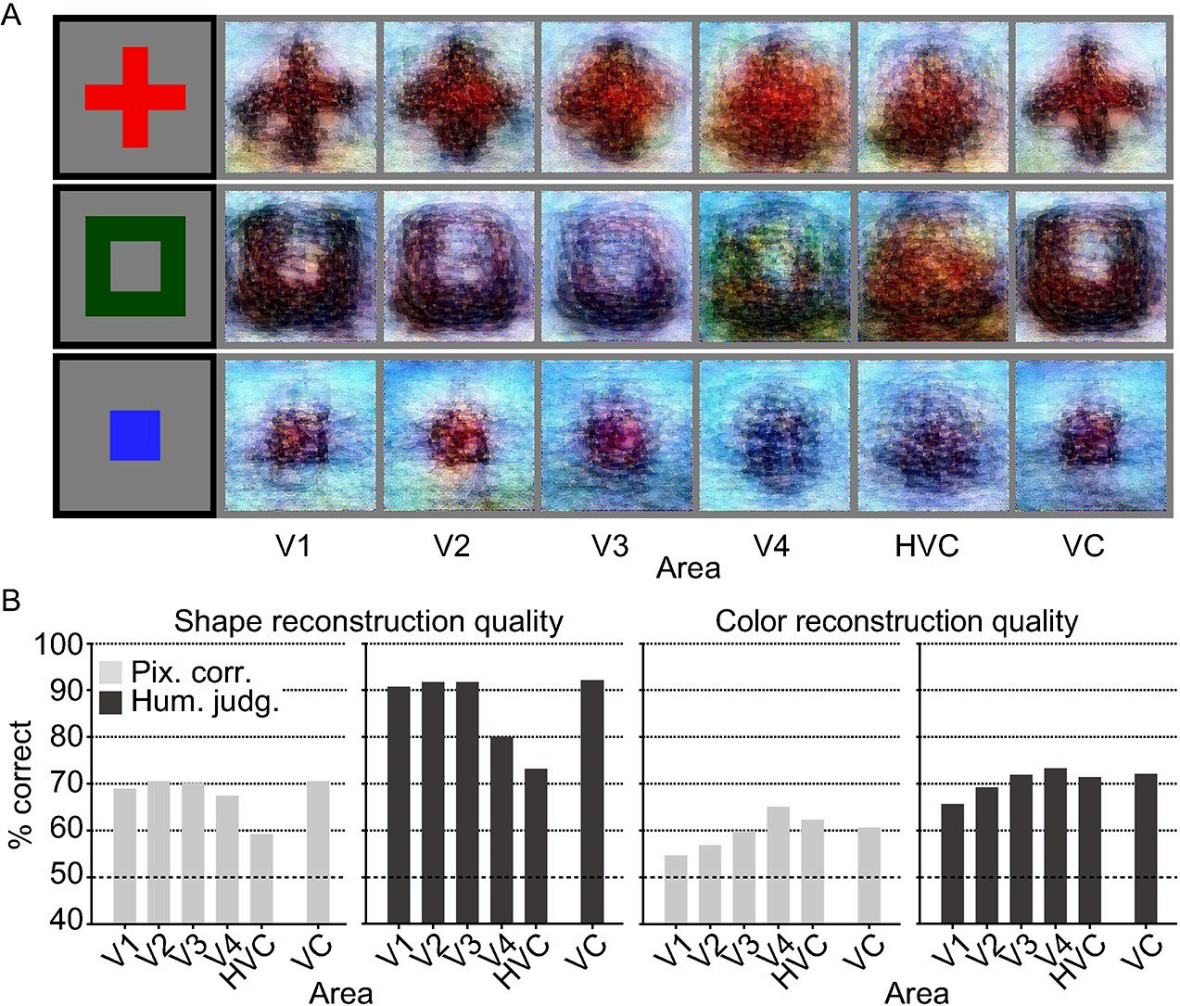
Reconstructions of shape and color from multiple visual areas. (**A**) Reconstructions of artificial shapes from multiple visual areas (DNN 1–8, without the DGN). The black and gray surrounding frames indicate presented and reconstructed images respectively. (**B**) Reconstruction quality of shape and color for different visual areas (three subjects pooled, *N* = 120; chance level, 50%).

To assess how the shapes and colors of the stimulus images were reconstructed, we separately evaluated the reconstruction quality of each of shape and color by comparing reconstructed images of the same colors and shapes. Analyses with different visual areas showed different trends in reconstruction quality for shapes and colors (Fig 7A and see S14 Fig for more examples). Human judgment evaluations suggested that shapes were reconstructed better from early visual areas, whereas colors were reconstructed better from the mid-level visual area V4 (Fig 7B and see S15 Fig for individual subjects; ANOVA, interaction between task type [shape vs. color] and brain areas [V1 vs. V4], *P* < 0.01), although the interaction effect was marginal when considering evaluations by pixel-wise spatial correlation (*P* = 0.06). These contrasting patterns further support the success of shape and color reconstructions and indicate that our method can be a useful tool to characterize the information content encoded in the activity patterns of individual brain areas by visualization.

**Fig 8:**
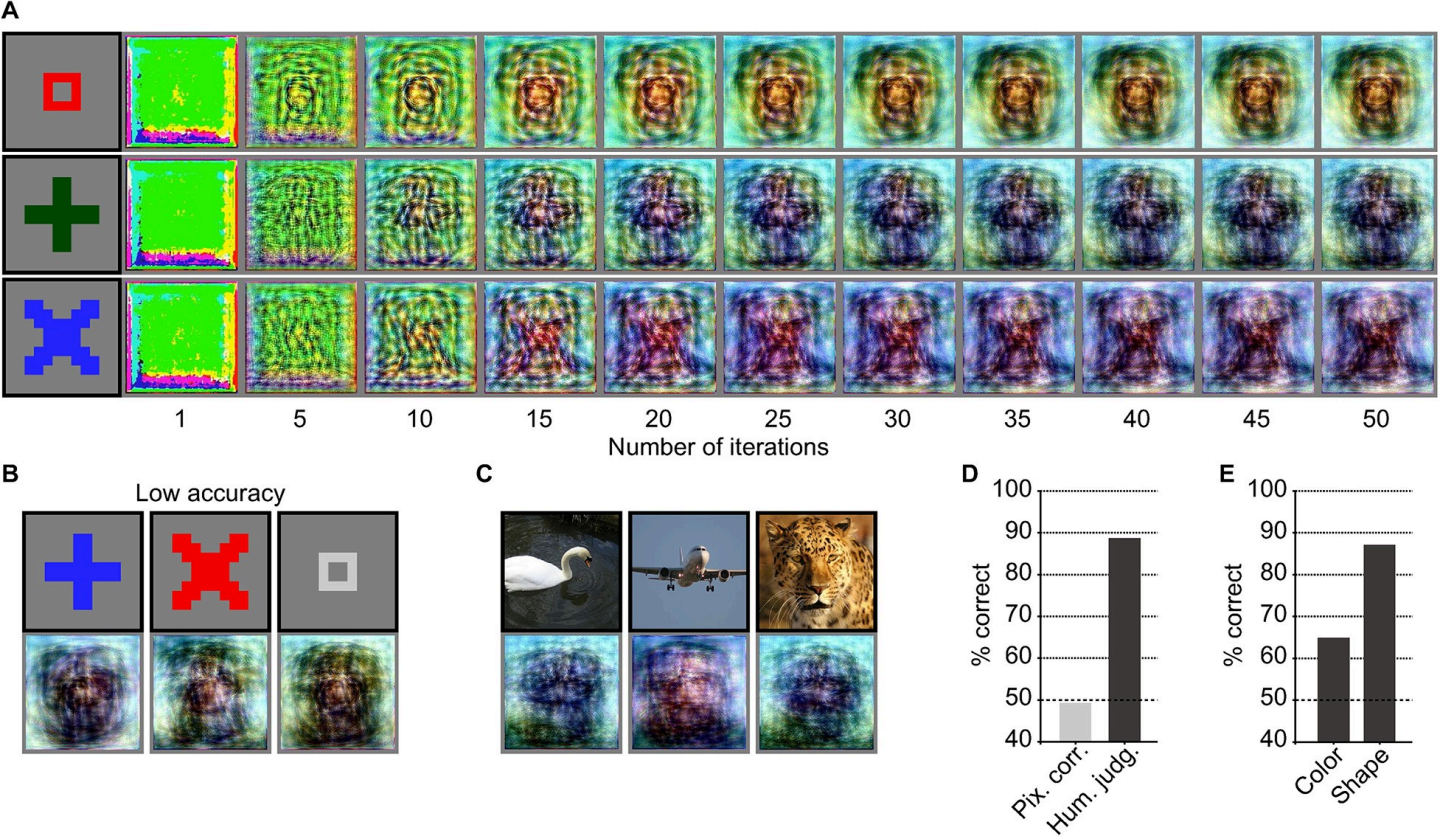
Imagery reconstructions. The black and gray surrounding frames indicate presented and reconstructed images respectively (VC activity, DNN 1–8, without the DGN). (**A**) Reconstructions for imagined artificial shapes through optimization processes. Reconstructed images obtained through the optimization processes are shown for images with high human judgment accuracy. (**B**) Reconstructions of imagined artificial shapes with low human judgment accuracy. (**C**) Reconstructions for imagined natural images. (**D**) Reconstruction quality of imagined artificial shapes (three subjects pooled, *N* = 45; chance level, 50%). (**E**) Reconstruction quality of imagined artificial shapes separately evaluated for color and shape by human judgment (three subjects pooled, *N* = 45; chance level, 50%).

Finally, to explore the possibility of visually reconstructing subjective content, we performed an experiment in which participants were asked to produce mental imagery of natural and artificial images shown prior to the task session. The reconstructions generated from brain activity due to mental imagery are shown in Fig 8 (see S16 Fig and S3 Movie for more examples). While the reconstruction quality varied across subjects and images, rudimentary reconstructions were obtained for some of the artificial shapes (Fig 8A and 8B for high and low accuracy images, respectively). In contrast, imagined natural images were not well reconstructed, possibly because of the difficulty of imagining complex natural images (Fig 8C; see S17 Fig for vividness scores of imagery). While the pixel-wise spatial correlation evaluations of reconstructed artificial images did not show high accuracy (Fig 8D; 51.9%; see S18 Fig for individual subjects), this may have been due to the possible disagreements in positions, colors and luminance between target and reconstructed images. Meanwhile, the human judgment evaluations showed accuracy higher than the chance level, suggesting that imagined artificial images were recognizable from the reconstructed images (Fig 8D; 83.2%; one-sided signed-rank test, *P* < 0.01; see S18 Fig for individual subjects). Furthermore, separate evaluations of color and shape reconstructions of artificial images suggested that shape rather than color had a major contribution to the high proportion of correct answers by human raters (Fig 8E; color, 64.8%; shape, 87.0%; two-sided signed-rank test, *P* < 0.01; see S19 Fig for individual subjects). Additionally, poor but sufficiently recognizable reconstructions were obtained even from brain activity patterns in the primary visual area (V1; 63.8%; three subjects pooled; one-sided signed-rank test, *P* < 0.01; see S20 Fig for reconstructed images and S21 Fig and S22 Fig for quantitative evaluations), possibly supporting the notion that low-level visual features are encoded in early visual cortical activity during mental imagery [21]. Taken together, these results provide evidence for the feasibility of visualizing imagined content from brain activity patterns.

## Discussion

We have presented a novel approach to reconstruct perceptual and mental content from human brain activity combining visual features from the multiple layers of a DNN. We successfully reconstructed viewed natural images, especially when combined with a DGN. The results from the extensive analysis on each subject were replicated across different subjects. Reconstruction of artificial shapes was also successful, even though the reconstruction models used were trained only on natural images. The same method was also applied to mental imagery, and revealed rudimentary reconstructions of mental content.

Our method is capable of reconstructing various types of images, including natural images, colored artificial shapes, and alphabetical letters, even though each component of our reconstruction model, the DNN models and the DNN feature decoders, was solely trained with natural images. The results strongly demonstrated that our method was certainly able to ‘reconstruct’ or ‘generate’ images from brain activity, differentiating our method from the previous attempts to visualize perceptual contents using the exemplar matching approach, which suffers from restrictions derived from pre-selected image/movie sets [3, 4].

We introduced the GAN-based constraint using the DGN for natural image reconstructions to enhance the naturalness of reconstructed images, rendering semantically meaningful details to the reconstructions. A variant of the GAN-based approach has demonstrated the utility in a previous face image reconstruction study, too [22]. GAN-derived feature space appears to provide efficient constraints on resultant images to enhance the perceptual resemblance to the image set on which a GAN is trained.

While one of the strengths of the present method is its generalizability across image types, there remains room for substantial improvements in reconstruction performance. Because we used the models (DNNs and decoders) trained with natural ‘object’ images from the ImageNet database [14], whose images contain objects around the center, it would not be optimal for the reconstruction of other types of images. Furthermore, because we used the DNN model trained to classify images into 1,000 object categories, the representations acquired in the DNN would be specifically suited to the particular objects. One could train the models with diverse types of images, such as scenes, textures, and artificial shapes, as well as object images, to improve general reconstruction performance. If the target image type is known in prior, one can use a specific set of images and a DNN model training task that are matched to it.

Other DNN models with different architectures could also be used to improve general reconstruction performance. As the reconstruction quality is positively correlated with the feature decoding accuracy (S9 Fig), DNNs with highly decodable units are likely to improve reconstructions. Recent studies evaluated different types of DNNs in term of the prediction accuracy of brain activity given their feature values (or the encoding accuracy) [23–25]. Although it remains to be seen how closely the encoding and decoding accuracies are linked, it is expected that more ‘brain-like’ DNN models would yield high-quality reconstructions.

Our approach provides a unique window into our internal world by translating brain activity into images via hierarchical visual features. Our method can also be extended to decode mental contents other than visual perception and imagery. By choosing an appropriate DNN architecture with substantial homology with neural representations, brain-decoded DNN features could be rendered into movies, sounds, text, or other forms of sensory/mental representations. The externalization of mental contents by this approach might prove useful in communicating our internal world via brain–machine/computer interfaces.

## Materials and methods

### Ethics statement

All subjects provided written informed consent for participation in our experiments, in accordance with the Declaration of Helsinki, and the study protocol was approved by the Ethics Committee of ATR.

### Subjects

Three healthy subjects with normal or corrected-to-normal vision participated in our experiments: Subject 1 (male, age 33), Subject 2 (male, age 23) and Subject 3 (female, age 23). This sample size was chosen on the basis of previous fMRI studies with similar experimental designs [1, 10].

### Visual stimuli

Visual stimuli consisted of natural images, artificial shapes, and alphabetical letters. The natural images were identical to those used in Horikawa & Kamitani (2017) [10], which were originally collected from the online image database *ImageNet* (2011, fall release) [14]. The images were cropped to the center and resized to 500 × 500 pixels. The artificial shapes consisted of a total of 40 combinations of 5 shapes and 8 colors (red, green, blue, cyan, magenta, yellow, white, and black), in which the shapes were identical to those used in Miyawaki et al. (2008) [1] and the luminance was matched across colors except for white and black. The alphabetical letter images consisted of the 10 black letters, A, C, E, I, N, O, R, S, T, and U.

### Experimental design

We conducted two types of experiments: image presentation experiments and a mental imagery experiment. The image presentation experiments consisted of four distinct session types, in which different variants of visual images were presented (training natural images, test natural images, artificial shapes, and alphabetical letters). All visual stimuli were rear-projected onto a screen in the fMRI scanner bore using a luminance-calibrated liquid crystal display projector. To minimize head movements during fMRI scanning, subjects were required to fix their heads using a custom-molded bite-bar individually made for each subject. Data from each subject were collected over multiple scanning sessions spanning approximately 10 months. On each experimental day, one consecutive session was conducted for a maximum of 2 hours. Subjects were given adequate time for rest between runs (every 5–8 min) and were allowed to take a break or stop the experiment at any time.

### Image presentation experiment

The image presentation experiments consisted of four distinct types of sessions: training natural-image sessions, test natural-image sessions, artificial-shape sessions, and alphabetical-letter sessions. Each session consisted of 24, 24, 20, and 12 separate runs, respectively. For these four sessions, each run comprised 55, 55, 44, and 11 stimulus blocks, respectively, with these consisting of 50, 50, 40, and 10 blocks with different images, and 5, 5, 4, and 1 randomly interspersed repetition blocks where the same image as in the previous block was presented (7 min 58 s for the training and test natural-image sessions, 6 min 30 s for the artificial-shape sessions, and 5 min 2 s for the alphabetical-letter sessions, for each run). Each stimulus block was 8 s (training natural-images, test natural-images, and artificial-shapes) or 12 s (alphabetical-letters) long, and was followed by a 12-s rest period for the alphabetical-letters, while no rest period was used for the training natural-images, test natural-images, and artificial-shapes. Images were presented at the center of the display with a central fixation spot and were flashed at 2 Hz (12 × 12 and 0.3 × 0.3 degrees of visual angle for the visual images and fixation spot respectively). The color of the fixation spot changed from white to red for 0.5 s before each stimulus block began, to indicate the onset of the block. Additional 32- and 6-s rest periods were added to the beginning and end of each run respectively. Subjects were requested to maintain steady fixation throughout each run and performed a one-back repetition detection task on the images, responding with a button press for each repeated image, to ensure they maintained their attention on the presented images (mean task performance across three subjects: sensitivity 0.9820; specificity 0.9995; pooled across sessions). In one set of training natural-image session, a total of 1,200 images were presented only once. This set of training natural-image session was repeated five times (1,200 × 5 = 6,000 samples for training). In the test natural-image, artificial-shape, and alphabetical-letter sessions, 50, 40, and 10 images were presented 24, 20, and 12 times each respectively. The presentation order of the images was randomized across runs.

### Mental imagery experiment

In the mental imagery experiment, subjects were required to visually imagine (recall) one of 25 images selected from those presented in the test natural image and artificial shape sessions of the image presentation experiment (10 natural images and 15 artificial images). Prior to the experiment, subjects were asked to relate words to visual images, so that they could recall the visual images from word cues. The imagery experiment consisted of 20 separate runs, with each run containing 26 blocks (7 min 34 s for each run). The 26 blocks consisted of 25 imagery trials and a fixation trial, in which subjects were required to maintained a steady fixation without any imagery. Each imagery block consisted of a 4-s cue period, an 8-s mental imagery period, a 3-s evaluation period, and a 1-s rest period. Additional 32- and 6-s rest periods were added to the beginning and end of each run respectively. During the rest periods, a white fixation spot was presented at the center of the display. At 0.8 s before each cue period, the color of the fixation spot changed from white to red for 0.5 s, to indicate the onset of the blocks. During the cue period, words specifying the visual images to be imagined were visually presented around the center of the display (1 target and 25 distractors). The position of each word was randomly changed across blocks to avoid cue-specific effects contaminating the fMRI response during mental imagery periods. The word corresponding to the image to be imagined was presented in red (target) and the other words were presented in black (distractors). Subjects were required to start imagining a target image immediately after the cue words disappeared. The imagery period was followed by a 3-s evaluation period, in which the word corresponding to the target image and a scale bar was presented, to allow the subjects to evaluate the correctness and vividness of their mental imagery on a five-point scale (very vivid, fairly vivid, rather vivid, not vivid, cannot correctly recognize the target). This was performed by pressing the left and right buttons of a button box placed in their right hand, to change the score from its random initial setting. As an aid for remembering the associations between words and images, the subjects were able to use control buttons to view the word and visual image pairs during every inter-run-rest period.

### MRI acquisition

fMRI data were collected using a 3.0-Tesla Siemens MAGNETOM Verio scanner located at the Kokoro Research Center, Kyoto University. An interleaved T2*-weighted gradient-echo echo planar imaging (EPI) scan was performed to acquire functional images covering the entire brain (TR, 2000 ms; TE, 43 ms; flip angle, 80 deg; FOV, 192 × 192 mm; voxel size, 2 × 2 × 2 mm; slice gap, 0 mm; number of slices, 76; multiband factor, 4). High-resolution anatomical images of the same slices obtained for the EPI were acquired using a T2-weighted turbo spin echo sequence (TR, 11000 ms; TE, 59 ms; flip angle, 160 deg; FOV, 192 × 192 mm; voxel size, 0.75 × 0.75 × 2.0 mm). T1-weighted magnetization-prepared rapid acquisition gradient-echo (MP-RAGE) fine-structural images of the entire head were also acquired (TR, 2250 ms; TE, 3.06 ms; TI, 900 ms; flip angle, 9 deg, FOV, 256 × 256 mm; voxel size, 1.0 × 1.0 × 1.0 mm).

### MRI data preprocessing

The first 8 s of scans from each run were discarded to avoid MRI scanner instability effects. We then used SPM (http://www.fil.ion.ucl.ac.uk/spm) to perform three-dimensional motion correction on the fMRI data. The motion-corrected data were then coregistered to the within-session high-resolution anatomical images with the same slices as the EPI, and then subsequently to the whole-head high-resolution anatomical images. The coregistered data were then re-interpolated to 2 × 2 × 2 mm voxels.

Data samples were created by first regressing out nuisance parameters from each voxel amplitude for each run, including any linear trend and the temporal components proportional to the six motion parameters calculated during the motion correction procedure. After that, voxel amplitudes were normalized relative to the mean amplitude of the initial 24-s rest period of each run and were despiked to reduce extreme values (beyond ± 3 SD for each run). The voxel amplitudes were then averaged within each 8-s (training natural image-sessions) or 12-s (test natural-image, artificial-shape, and alphabetical-letter sessions) stimulus block (four or six volumes), and within the 16-s mental imagery block (eight volumes, mental imagery experiment), after shifting the data by 4 s (two volumes) to compensate for hemodynamic delays.

### Regions of interest (ROI)

V1, V2, V3, and V4 were delineated following the standard retinotopy experiment [26, 27]. The lateral occipital complex (LOC), fusiform face area (FFA), and parahippocampal place area (PPA) were identified using conventional functional localizers [28–30] (See S1 Supporting Information for details). A contiguous region covering the LOC, FFA, and PPA was manually delineated on the flattened cortical surfaces, and the region was defined as the higher visual cortex (HVC). Voxels overlapping with V1–V3 were excluded from the HVC. Voxels from V1–V4 and the HVC were combined to define the visual cortex (VC). In the regression analysis, voxels showing the highest correlation coefficient with the target variable in the training image session were selected to decode each feature (with a maximum of 500 voxels).

### Deep neural network features

We used the *Caffe* implementation of the *VGG19* deep neural network (DNN) model [13], which was pre-trained with images in *ImageNet* [14] to classify 1,000 object categories (the pre-trained model is available from https://github.com/BVLC/caffe/wiki/Model-Zoo). The VGG19 model consisted of a total of sixteen convolutional layers and three fully connected layers. To compute outputs by the VGG19 model, all visual images were resized to 224 × 224 pixels and provided to the model. The outputs from the units in each of the 19 layers (immediately after convolutional or fully connected layers, before rectification) were treated as a vector in the following decoding and reconstruction analysis. The number of units in each of the19 layers is the following: conv1_1 and conv1_2, 3211264; conv2_1 and conv2_2, 1605632; conv3_1, conv3_2, conv3_3, and conv3_4, 802816; conv4_1, conv4_2, conv4_3, and conv4_4, 401408; conv5_1, conv5_2, conv5_3, and conv5_4, 100352; fc6 and fc7, 4096; and fc8, 1000. In this study, we named five groups of convolutional layers as DNN1–5 (DNN1: conv1_1, and conv1_2; DNN2: conv2_1, and conv2_2; DNN3: conv3_1, conv3_2, conv3_3, and conv3_4; DNN4: conv4_1, conv4_2, conv4_3, and conv4_4; and DNN5: conv5_1, conv5_2, conv5_3, and conv5_4), and three fully-connected layers as DNN6–8 (DNN6: fc6; DNN7: fc7; and DNN8: fc8). We used the original pre-trained VGG19 model to compute the feature unit activities, but for analyses with fMRI data from the mental imagery experiment, we changed the DNN model so that the max pooling layers were replaced by average pooling layers, and the ReLU activation function was replaced by a leaky ReLU activation function with a negative slope of 0.2 (see Simonyan & Zisserman (2015) [13] for the details of the original DNN architecture).

### DNN feature decoding analysis

We used a set of linear regression models to construct multivoxel decoders to decode the DNN feature vector of a seen image from the fMRI activity patterns obtained in the training natural-image sessions (training dataset). In this study, we used the sparse linear regression algorithm (SLR) [31], which can automatically select important voxels for decoding by introducing sparsity into a weight estimation through Bayesian estimation of parameters with the automatic relevance determination (ARD) prior (see Horikawa & Kamitani (2017) [10] for a detailed description). The training dataset was used to train the decoders to decode the values of individual units in the feature vectors of all DNN layers (one decoder for one DNN feature unit), and the trained decoders were then applied to the test datasets. For details of the general procedure of feature decoding, see Horikawa & Kamitani (2017) [10].

For the test datasets, fMRI samples corresponding to the same stimulus or mental imagery were averaged across trials to increase the signal-to-noise ratio of the fMRI signals. To compensate for possible differences in the signal-to-noise ratio between training and test samples, the decoded features of individual DNN layers were normalized by multiplying them by a single scalar, so that the norm of the decoded vectors of individual DNN layers matched with the mean norm of the true DNN feature vectors computed from independent 10,000 natural images. This norm-corrected vector was then subsequently provided to the reconstruction algorithm (See Supporting Information for details of the norm-correction procedure).

### Reconstruction from a single DNN layer

Given a DNN feature vector decoded from brain activity, an image was generated by solving the following optimization problem [11].

$$v^{*}=\underset{v}{\mathrm{argmin}}\frac{1}{2}\sum_{i=1}^{I_{l}}{\left(\phi_{i}^{\left(l\right)}(v)-y_{i}^{\left(l\right)}\right)}^{2}$$ (1)

$$=\underset{v}{\mathrm{argmin}}\frac{1}{2}{\left\|\Phi^{\left(l\right)}(v)-y^{\left(l\right)}\right\|}_{2}^{2}$$ (2)

where $v\in R^{224\times 224\times 3}$ is a vector whose elements are pixel values of an image (224 × 224 × 3 corresponds to height × width × RGB color channel), and v* is the reconstructed image. $\phi_{i}^{\left(l\right)}:\;R^{224\times 224\times 3}\to R$ is the feature extraction function of the *i*-th DNN feature in the *l*-th layer, with $\phi_{i}^{\left(l\right)}(v)$ being the output value from the *i*-th DNN unit in the *l*-th layer for the image **v**. *I*_*l*_ is the number of units in the *l*-th layer, and $y_{i}^{\left(l\right)}$ is the value decoded from brain activity for the *i*-th feature in the *l*-th layer. For simplicity, the same cost function was rewritten with a vector function in the second line. $\Phi^{\left(l\right)}:\;R^{224\times 224\times 3}\to R^{I_{l}}$ is the function whose *i*-th element is $\phi_{i}^{\left(l\right)}$ and $y^{\left(l\right)}\in R^{I_{l}}$ is the vector whose *i*-th element is $y_{i}^{\left(l\right)}$.

The above cost function was minimized by either a limited-memory BFGS algorithm (L-BFGS) [32–34] or by a gradient descent with momentum algorithm [35], with L-BFGS being used unless otherwise stated. The obtained solution was taken to be the image reconstructed from the brain activity (see Supporting Information for details of optimization methods).

### Reconstruction from multiple DNN layers

To combine the DNN features from multiple layers, we took a weighted sum of the cost functions for individual DNN layers, given by

$$v^{*}=\underset{v}{\mathrm{argmin}}\frac{1}{2}\sum_{l\in L}\beta_{l}{\left\|\Phi^{\left(l\right)}(v)-y^{\left(l\right)}\right\|}_{2}^{2}$$ (3)

where *L* is a set of DNN layers and *β*_*l*_ is a parameter that determines the contribution of the *l*-th layer. We set *β*_*l*_ to $1/{\left\|y^{\left(l\right)}\right\|}_{2}^{2}$ to balance the contributions of individual DNN layers. This cost function was minimized by the L-BFGS algorithm. The DNN layers included in *L* were combined. In the main analyses, we combined all convolutional (DNN1–5) and fully connected layers (DNN6–8), unless otherwise stated.

### Natural image prior

To improve the ‘naturalness’ of reconstructed images, we modified the reconstruction algorithm by introducing a constraint. To constrain the resulting images from all possible pixel contrast patterns, we reduced the degrees of freedom by introducing a generator network derived using the generative adversarial network algorithm (GAN) [15], which has recently been shown to have good performance in capturing a latent space explaining natural images [16]. In the GAN framework, a set of two neural networks, which are called a generator and a discriminator, are trained. The generator is a function to map from a latent space to the data space (i.e. pixel space), and the discriminator is a classifier that predicts whether a given image is a sample from real natural images or an output from the generator. The discriminator is trained to increase its predictive power, while the generator is trained to decrease it. We considered constraining our reconstructed images to be in the subspace consisting of the images that could be produced by a generator trained to produce natural images [12, 36]. This is expressed by

$$z^{*}=\underset{z}{\mathrm{argmin}}\frac{1}{2}\sum_{l\in L}\beta_{l}{\left\|\Phi^{\left(l\right)}(G(z))-y^{\left(l\right)}\right\|}_{2}^{2}$$ (4)

and

$$v^{*}=G(z^{*}).$$ (5)

*G* is the generator, as the mapping function from the latent space to the image space, which we have called a deep generator network (DGN). In our reconstruction analysis, we used a pre-trained DGN which was provided by Dosovitskiy & Brox (2016; available from https://github.com/dosovits/caffe-fr-chairs; trained model for fc7) [36].

The above cost function for the reconstruction with respect to **z** was minimized by gradient descent with momentum. We used the zero vector as the initial value. To keep **z** within a moderate range, we restricted the range of each element of **z** following the method of a previous study [36].

### Evaluation of reconstruction quality

Reconstruction quality was evaluated by either objective or subjective assessment [5, 17, 18]. For the objective assessment, we performed a pairwise similarity comparison analysis, in which a reconstructed image was compared with two candidate images (its original image and a randomly selected image), to test whether its pixel-wise spatial correlation coefficient (Pearson correlation between vectorized pixel values) with the original image was higher than that for a randomly selected image. For the subjective assessment, we conducted a behavioral experiment with a group of 13 raters (5 females and 8 males, aged between 19 and 37 years). On each trial of the experiment, the raters viewed a display presenting a reconstructed image (at the bottom) and two candidate images (displayed at the top; the original image and a randomly selected image), and were asked to select the candidate image most similar to the reconstructed one presented at the bottom. Each trial continued until the raters made a response. For both types of assessments, the proportion of trials, in which the original image was selected as more similar was calculated as a quality measure. In both objective and subjective assessments, each reconstructed image was tested with all pairs of the images from the same types of images (natural-images, artificial-shapes, and alphabetical-letters for images from the image presentation sessions, and natural-images and artificial-shapes for images from the mental imagery session; e.g., for the test natural-images, one of the 50 reconstructions was tested with 49 pairs, with each one consisting of one original image and another image from the rest of 49, resulting in 50 × 49 = 2,450 comparisons). The quality of an individual reconstructed image was evaluated by the percentage of correct answers that was calculated as the proportion of correct trials among all trials where the reconstructed image was tested (i.e., a total of 49 trials for each one of the test natural-images). The resultant percentages of correct answers were then used for the following statistical tests.

To compare the reconstruction quality across different combinations of DNN layers, we also used objective and subjective assessments. For the subjective assessment, we conducted another behavioral experiment with another group of 7 raters (2 females and 5 males, aged between 20 and 37 years). On each trial of the experiment, the raters viewed a display presenting one original image (at the top) and two reconstructed images of the same original image (at the bottom) obtained from different combinations of the DNN layers, and were asked to judge which of the two reconstructed images was better. This pairwise comparison was conducted for all pairs of the combinations of DNN layers (28 pairs), and for all stimulus images presented in the test natural-image session (50 samples). Each trial continued until the raters made a response. We calculated the proportion of trials, in which the reconstructed image obtained from a specific combination of DNN layers was judged as the better one, and then this value was treated as the winning percentage of this combination of DNN layers. For the objective assessment, the same pairwise comparison was conducted using pixel-wise spatial correlations, in which pixel-wise spatial correlations to the original image were compared between two combinations of DNN layers to judge the better combination of DNN layers. The results obtained from all test samples (50 samples from the test natural-image dataset) were used to calculate the winning percentage of each combination of DNN layers in the same manner with the subjective assessment.

These assessments were performed individually for each set of reconstructions from the different subjects and datasets (e.g., test natural-images from Subject 1). For the subjective assessments, one set of reconstructed images was tested with at least three raters. The evaluation results from different raters were averaged within the same set of reconstructions and were treated in the same manner as the evaluation results from the objective assessment.

### Statistics

We used two-sided signed-rank tests to examine differences in assessed reconstruction quality according to the different conditions (*N* = 150, 120, and 45 for the test-natural images, artificial shapes, and imagery images, respectively) and used ANOVA to examine interaction effects between task types and brain areas for artificial shapes (*F* (1,1) = 28.40 by human judgment; *F* (1,1) = 3.53 by pixel-wise spatial correlation). We used one-sided signed-rank tests to examine the significance of correct classification accuracy by the human judgment for evaluations of the imagery image reconstructions (*N* = 45).

## Supporting information

S1 Supporting Information Supplementary materials and methods. (PDF)

S1 Fig DNN feature decoding accuracy. DNN feature decoding accuracy obtained from VC activity was evaluated by the correlation coefficient between the true and decoded feature values of each feature unit following the procedure in Horikawa & Kamitani (2017) [10]. The evaluation was individually performed for each of the three types of seen images (natural images, artificial shapes, and alphabetical letters) and each of the two types imagery images (natural images and artificial shapes). Correlation coefficients were averaged across units in each DNN layer. The mean correlation coefficients are shown for each types of layers (error bars, 95% confidence interval [C.I.] across units; *N* of each layer equals to the number of units in each layer; see Material and Methods: “Deep neural network features” for details).
(PDF)

S2 Fig Examples of natural image reconstructions obtained with the DGN. The black and gray surrounding frames indicate presented and reconstructed images respectively (VC activity, DNN 1–8, with the DGN). The three columns of reconstructed images correspond to reconstructions from three subjects. For copyright reasons, we present only a subset of the 50 test natural images; those for which the copyright holders gave us permission to use.
(PDF)

S3 Fig Reconstructions from the generic object decoding dataset. The same reconstruction analysis was performed with a previously published dataset [10] (VC activity, DNN 1–8, with the DGN). See Horikawa & Kamitani (2017) [10] for details of the data. The black and gray surrounding frames indicate presented and reconstructed images respectively. The five columns of reconstructed images correspond to reconstructions from five subjects.
(PDF)

S4 Fig Reconstruction quality of seen natural images for individual subjects. Evaluations on individual subjects’ results are separately shown (VC activity; DNN1–8; *N* = 50; chance level, 50%; cf., Fig 3B), indicating that overall tendency was almost consistent across different subjects, except that the human judgment accuracy of reconstructions from Subject 3 showed slightly higher accuracy without the DGN than that with the DGN. Evaluations of reconstructions using pixel-wise spatial correlation for Subject 1–3 showed 78.4%, 74.2%, and 75.7% with the DGN, and 80.4%, 77.2%, and 81.3% without the DGN, respectively. Evaluations of reconstructions using human judgment for Subject 1–3 showed 98.5%, 97.3%, and 95.3% with the DGN, and 96.6%, 94.7%, and 96.7% without the DGN, respectively.
(PDF)

S5 Fig Other examples of natural image reconstructions obtained without the DGN. The black and gray surrounding frames indicate presented and reconstructed images respectively (VC activity, DNN 1–8, without the DGN). The three columns of reconstructed images correspond to reconstructions from three subjects.
(PDF)

S6 Fig Reconstructions from different initial states. The black and gray surrounding frames indicate presented and reconstructed images respectively (VC activity, DNN 1–8). We used different initial states for reconstructions with and without the DGN. For reconstructions with the DGN, we additionally performed the reconstruction analysis using a Gaussian-random-value vector (mean = 0, standard deviation = 1) as the initial state as well as the zero-value vector (main analysis; e.g., Fig 2). For reconstructions without the DGN, we also performed reconstructions from a uniform-random-value image (ranged between 0 and 255) and the zero-value image in addition to the spatially uniform image with the mean RGB values of natural images (main analysis; e.g., Fig 3). For comparison, reconstructed images from different initial states are compared within the same subjects. The results showed slightly different but almost equivalent images from different initial states, demonstrating the stability of our reconstructions.
(PDF)

S7 Fig Other examples of reconstructions with a variable number of multiple DNN layers. The black and gray surrounding frames indicate presented and reconstructed images respectively (VC activity, without the DGN).
(PDF)

S8 Fig Examples of reconstructions from individual DNN layers. The black and gray surrounding frames indicate presented and reconstructed images respectively (without the DGN). We used DNN features from individual layers (DNN1, DNN2, …, or DNN8) as well as the combination of all DNN layers (DNN1–8) for the reconstruction analysis, in which either of true or decoded features (VC activity) were provided to the reconstruction algorithm. While reconstructions from individual layers, especially from higher layers, showed poorer reconstruction quality even from true features, combining multiple layers produced almost complete reconstructions of original images from true features and good reconstructions from decoded features (cf., Fig 4 and S7 Fig).
(PDF)

S9 Fig Correlations between feature decoding accuracy and reconstruction quality. To investigate the relations between feature decoding accuracy and reconstruction quality, we first evaluated feature decoding accuracies for individual samples instead of those for individual DNN units (cf., S1 Fig; see Materials and Methods: “Evaluation of reconstruction quality” for how to evaluate the reconstruction quality for individual samples). To evaluate the feature decoding accuracy for each sample, Pearson’s correlation coefficients were caluculated between the decoded and true feature values for a single stimulus image using all units within each layer. To avoid estimating spuriously high correlations due to baseline and scale differences across units, feature values of each unit of the test data (test natural-image) were z-normalized using means and standard deviations estimated from the training data (training natural-image) before calculating correlations. Using the estimated feature decoding accuracy and reconstruction quality for individual samples (*N* = 50), Pearson’s correlation coefficients were further calculated between the reconstruction quality (VC activity; with or without the DGN, DNN1–8) and the feature decoding accuracy from individual layers or mean accuracy averaged across 19 layers. While the correlations varied across layers and subjects, the results on average showed positive correlations between the feature decoding accuracy and the reconstruction quality for all combinations of the assessments and the reconstruction algorithms, suggesting that higher decoding accuracy would lead to better reconstruction quality. Interestingly, the analysis showed distinct correlation patterns across layers between the two assessment types, showing that high correlaions were specifically observed from early layers with the pixel-wise spatial correlations although moderately high correlations were observed rather evenly from most layers with the human judgment. These results may reflect the different characteristics of the two assessments, indicating that the pixel-wise correlation is suited to evaluate accuracy in low-level features whereas the human judgment is capable of evaluating accuracy in multiple-levels of visual features.
(PDF)

S10 Fig All examples of artificial shape reconstructions. The black and gray surrounding frames indicate presented and reconstructed images respectively (VC activity, DNN 1–8, without the DGN). The three rows of reconstructed images correspond to reconstructions from three subjects.
(PDF)

S11 Fig All examples of alphabetical letter reconstructions. The black and gray surrounding frames indicate presented and reconstructed images respectively (VC activity, DNN 1–8, without the DGN). The three rows of reconstructed images correspond to reconstructions from three subjects.
(PDF)

S12 Fig Reconstruction quality of artificial shapes for individual subjects. Evaluations on individual subjects’ results are separately shown (VC activity; DNN1–8; without the DGN; *N* = 40; chance level, 50%; cf., Fig 6C left). Evaluations of reconstructions using pixel-wise spatial correlation showed 69.6%, 72.1%, and 69.8% for Subject 1–3, respectively. Evaluations of reconstructions using human judgment showed 91.7%, 91.3%, and 90.1% for Subject 1–3, respectively.
(PDF)

S13 Fig Reconstruction quality of alphabetical letters for individual subjects. Evaluations on individual subjects’ results are separately shown (VC activity; DNN1–8; without the DGN; *N* = 10; chance level, 50%; cf., Fig 6C right). Evaluations of reconstructions using pixel-wise spatial correlation showed 98.9%, 87.8%, and 100.0% for Subject 1–3, respectively. Evaluations of reconstructions using human judgment showed 100.0%, 98.9%, and 100.0% for Subject 1–3, respectively.
(PDF)

S14 Fig All examples of artificial shape reconstructions obtained from different visual areas (Subject 1). The black and gray surrounding frames indicate presented and reconstructed images respectively (DNN 1–8, without the DGN).
(PDF)

S15 Fig Reconstruction quality of shape and color for different visual areas for individual subjects. Evaluations on individual subjects’ results are separately shown (DNN1–8; without the DGN; *N* = 40; chance level, 50%; cf., Fig 7B). Evaluations by pixel-wise correlations and human judgment both showed almost consistent tendency across different subjects, showing that shapes were reconstructed better from early visual areas, whereas colors were reconstructed better from relatively higher visual areas.
(PDF)

S16 Fig Other examples of imagery image reconstructions. The black and gray surrounding frames indicate presented and reconstructed images respectively (VC activity, DNN 1–8, without the DGN). The three rows of reconstructed images correspond to reconstructions from three subjects. The rightmost images in the bottom row show reconstructions during maintenance of fixation without imagery.
(PDF)

S17 Fig Vividness scores for imagery images reported by subjects. Vividness scores reported during the imagery experiment are shown in descending order of mean vividness scores across trials for individual images. For each subject, the vividness scores were averaged across trials for the same imagery images (*N* = 20). For the pooled results, to eliminate baseline and variability differences across subjects, the vividness scores obtained from individual subjects were first converted to z-scores within each subject, and then averaged across all trials from three subjects (*N* = 60). The rightmost two bars indicated as “artificial” and “natural” show mean vividness scores separately pooled for artificial shapes (15 artificial shapes) and natural images (10 natural images). Error bars indicate 95% confidence intervals across trials.
(PDF)

S18 Fig Reconstruction quality of imagined artificial shapes for individual subjects. Evaluations on individual subjects’ results are separately shown (VC activity; DNN 1–8; without the DGN; *N* = 15; chance level, 50%; cf., Fig 8D). Evaluations of reconstructions using pixel-wise spatial correlation showed 49.5%, 52.4%, and 53.8% for Subject 1–3, respectively. Evaluations of reconstructions using human judgment showed 85.6%, 84.4%, and 79.5% for Subject 1–3, respectively.
(PDF)

S19 Fig Reconstruction quality of imagined artificial shapes for individual subjects separately evaluated for color and shape by human judgment. Evaluations on individual subjects’ results are separately shown (VC activity; DNN 1–8; without the DGN; *N* = 15; chance level, 50%; cf., Fig 8E). Evaluations of reconstructions with respect to color showed 71.1%, 56.7%, and 66.7% for Subject 1–3, respectively. Evaluations of reconstructions with respect to shape showed 91.1%, 88.9%, and 81.1% for Subject 1–3, respectively.
(PDF)

S20 Fig Imagery image reconstructions from V1. The black and gray surrounding frames indicate presented and reconstructed images respectively (V1 activity, DNN 1–8, without the DGN). The three rows of reconstructed images correspond to reconstructions from three subjects. The rightmost images in the bottom row show reconstructions during maintenance of fixation without imagery.
(PDF)

S21 Fig Reconstruction quality of imagined artificial shapes (reconstructed from V1). Evaluations on individual subjects’ results and their pooled result are separately shown (V1 activity; DNN 1–8; without the DGN; *N* = 15 for individual subjects and *N* = 45 for the pooled result; chance level, 50%; cf., Fig 8D). Evaluations of reconstructions using pixel-wise spatial correlation showed 48.2%, 51.3%, 48.4%, and 48.8% for Subject 1–3 and the pooled result, respectively. Evaluations of reconstructions using human judgment showed 57.7%, 73.5%, 60.1%, and 63.8% for Subject 1–3 and the pooled result, respectively.
(PDF)

S22 Fig Reconstruction quality of imagined artificial shapes separately evaluated for color and shape by human judgment (reconstructed from V1). Evaluations on individual subjects’ results and their pooled result are separately shown (V1 activity; DNN 1–8; without the DGN; *N* = 15 for individual subjects and *N* = 45 for the pooled result; chance level, 50%; cf., Fig 8E). Evaluations of reconstructions with respect to color showed 60.0%, 56.7%, 55.6%, and 57.4% for Subject 1–3 and the pooled result, respectively. Evaluations of reconstructions with respect to shape showed 63.9%, 77.8%, 63.3%, and 68.3% for Subject 1–3 and the pooled result, respectively. As shown with the reconstructed images from VC (cf., Fig 8E), separate evaluations of color and shape reconstructions of artificial images from V1 also showed that shape rather than color had a major contribution to the high proportion of correct answers by human raters (three subjects pooled; two-sided signed-rank test, *P* < 0.05).
(PDF)

S1 Movie Deep image reconstruction: Natural images. The iterative optimization process is shown (left, presented images; right, reconstructed images).
(MOV)

S2 Movie Deep image reconstruction: Artificial shapes. The iterative optimization process is shown (left, presented images; right, reconstructed images).
(MOV)

S3 Movie Deep image reconstruction: Imagery images. The iterative optimization process is shown (left, imagined images; right, reconstructed images).
(MOV)
