# Supplementary material for: Deep image reconstruction from human brain activity
Source: PLoS Comput Biol. 2019 Jan 14;15(1):e1006633. doi: 10.1371/journal.pcbi.1006633 (PMC6347330; doi:10.1371/journal.pcbi.1006633)
Supplement: S1 Supporting Information — (PDF) [file pcbi.1006633.s001.pdf]

Supporting Information for

Deep image reconstruction from human brain activity

Guohua Shen¶, Tomoyasu Horikawa¶, Kei Majima¶, Yukiyasu Kamitani*

* Corresponding Author

E-mail: kamitani@i.kyoto-u.ac.jp (YK)

¶These authors contributed equally to this work.

Supplementary Materials and Methods

Localizer experiments. Retinotopy and functional localizer experiments were conducted to identify the seven visual areas analyzed in the study.

The retinotopy experiments were conducted according to a conventional protocol [1, 2]. We used a rotating wedge and an expanding ring of a flickering checkerboard. The data were used to delineate the borders between visual cortical areas, and to identify the retinotopic map (V1–V4) on the flattened cortical surfaces of individual subjects.

We also performed functional localizer experiments to identify the lateral occipital complex (LOC) [3], fusiform face area (FFA) [4], and parahippocampal place area (PPA) [5] for each individual subject. The localizer experiment consisted of 8 runs, with each run containing 16 stimulus blocks. In this experiment, intact or scrambled images (12 × 12 degrees of visual angle) from face, object, house, and scene categories were presented in the center of the display. Each of eight stimulus types (four categories × two conditions) was presented twice per run. Each stimulus block consisted of a 15-s intact or scrambled stimulus presentation. The intact and scrambled stimulus blocks were presented successively (the order of the intact and scrambled stimulus blocks was random), followed by a 15-s rest period with a uniform gray background. Extra 33-s and 6-s rest periods were added to the beginning and end of each run, respectively. In each stimulus block, 20 different images of the same type were presented for 0.3 s, followed by an intervening blank screen of 0.4 s.

MRI acquisition for localizer experiments. fMRI data were collected using a 3.0-Tesla Siemens MAGNETOM Verio scanner located at the Kokoro Research Center, Kyoto University. An interleaved T2*-weighted gradient-echo echo planar imaging (EPI) scan was performed to acquire functional images covering the entire occipital lobe (retinotopy experiment: TR, 2000 ms; TE, 30 ms; flip angle, 80 deg; FOV, 192 × 192 mm; voxel size, 3 × 3 × 3 mm; slice gap, 0 mm; number of slices, 30) or the entire brain (localizer experiment: TR, 3000 ms; TE, 30 ms; flip angle, 80 deg; FOV, 192 × 192 mm; voxel size, 3 × 3 × 3 mm; slice gap, 0 mm; number of slices, 46). High-resolution anatomical images

of the same slices obtained for the EPI were acquired using a T2-weighted turbo spin echo sequence (retinotopy experiment: TR, 6000 ms; TE, 57 ms; flip angle, 160 deg; FOV, 192 × 192 mm; voxel size, 0.75 × 0.75 × 3.0 mm; localizer experiments: TR, 7020 ms; TE, 69 ms; flip angle, 160 deg; FOV, 192 × 192 mm; voxel size, 0.75 × 0.75 × 3.0 mm).

MRI data preprocessing for data from the localizer experiments. The first 8-s of scans for experiments with TR = 2 s (retinotopy experiments) and 9-s of scans for experiments with TR = 3 s (localizer experiment) were discarded from each run to avoid MRI scanner instability effects. We then used SPM (http://www.fil.ion.ucl.ac.uk/spm) to perform three-dimensional motion correction on the fMRI data. The motion-corrected data were then coregistered to the within-session high-resolution anatomical images with the same slices as the EPI, and then subsequently to the whole-head high-resolution anatomical images. The coregistered data were then re-interpolated to 2 × 2 × 2 mm voxels.

Region of interest (ROI) selection. V1, V2, V3, and V4 were identified using the data from the retinotopy experiments [1, 2]. LOC, FFA, and PPA were identified using the data from the functional localizer experiments [3–5]. The data from the retinotopy experiment were transformed into Talairach space and the visual cortical borders were delineated on flattened cortical surfaces using BrainVoyager QX (http://www.brainvoyager.com; RRID: SCR_013057). The coordinates of voxels around the gray-white matter boundary in V1–V4 were identified and transformed back into the original coordinates of the EPI images. The localizer experiment data were analyzed using SPM. The voxels showing significantly higher activation in response to intact object, face, or scene images in comparison with scrambled images (two sided t-test, uncorrected $P < 0.05$ or 0.01) were identified, and defined as LOC, FFA, and PPA respectively. A contiguous region covering the LOC, FFA, and PPA was manually delineated on the flattened cortical surfaces, and the region was defined as the higher visual cortex (HVC). Voxels from V1–V4 and the HVC were combined to define the visual cortex (VC). In the regression analysis, voxels showing the highest correlation

coefficient with the target variable in the training image session were selected to decode each feature (with a maximum of 500 voxels).

Norm correction for decoded DNN feature vectors. Before reconstruction analysis, the DNN feature vector decoded from a given fMRI sample was multiplied by a scalar to match its norm to the mean across natural images.

$$\mathbf{y}_{rescaled} = \mathbf{y}_{raw} \frac{\mathbb{E}_{\mathbf{v}}\left[f\left(\boldsymbol{\phi}(\mathbf{v})\right)\right]}{f(\mathbf{y}_{raw})} \tag{1}$$

where $\mathbf{y}_{raw}$ is a decoded feature vector, and $\mathbf{y}_{rescaled}$ is the feature vector after the norm-correction. $\mathbf{v}$ is a vector whose elements are pixel values of an image, and $\boldsymbol{\phi}$ is the feature extraction function whose input is an image vector and output is the DNN feature vector for the input image. f is a function whose definition is given later, and $\mathbb{E}_{\mathbf{v}}\left[f\left(\boldsymbol{\phi}(\mathbf{v})\right)\right]$ denotes the expectation of $f\left(\boldsymbol{\phi}(\mathbf{v})\right)$ with respect to $\mathbf{v}$ across natural images. The expectation was calculated using 10,000 natural images randomly selected from the *ImageNet* database (2011, fall release) [6].

When the input of the function f is a DNN feature vector from a convolutional layer, we first calculate the standard deviation of the feature value across the units in each channel, and then the mean of this standard deviation across all channels is treated as the output value of f. When the input is a DNN feature vector from a fully-connected layer, the standard deviation of the feature value across the units in the layer is treated as the output value of f.

If f is the vector norm, our norm-correction exactly matches the given decoded vector with the mean norm across natural images. In this study, we adopted the definition of f explained above, because our norm-correction procedure led to slightly better reconstructions compared with the exact norm matching used in the early stages of the analysis, which were performed using independent preliminary data.

Optimization methods for reconstruction. The cost function for our reconstruction was minimized by limited-memory BFGS (L-BFGS) [7–9], or by gradient descent with momentum [10]. Each of these algorithms is explained in this section.

Given a DNN feature vector decoded from brain activity, an image was generated by solving the following optimization problem [11].

$$\mathbf{v}^* = \underset{\mathbf{v}}{\operatorname{argmin}} \frac{1}{2} \sum_{i=1}^{I_l} \left(\phi_i^{(l)}(\mathbf{v}) - y_i^{(l)} \right)^2 \tag{2}$$

$$= \underset{\mathbf{v}}{\operatorname{argmin}} \frac{1}{2} \left\| \mathbf{\Phi}^{(l)}(\mathbf{v}) - \mathbf{y}^{(l)} \right\|_2^2 \tag{3}$$

where $\mathbf{v} \in \mathbb{R}^{224 \times 224 \times 3}$ is a vector whose elements are pixel values of an image (224 × 224 × 3 corresponds to height × width × RGB color channel), and $\mathbf{v}^*$ is the reconstructed image. $\phi_i^{(l)}: \mathbb{R}^{224 \times 224 \times 3} \rightarrow \mathbb{R}$ is the feature extraction function of the *i*-th DNN feature in the *l*-th layer, with $\phi_i^{(l)}(\mathbf{v})$ being the output value from the *i*-th DNN unit in the *l*-th layer for the image $\mathbf{v}$. I_l is the number of units in the *l*-th layer, and $y_i^{(l)}$ is the value decoded from brain activity for the *i*-th feature in the *l*-th layer. For simplicity, the same cost function was rewritten with a vector function in the second line. $\mathbf{\Phi}^{(l)}: \mathbb{R}^{224 \times 224 \times 3} \rightarrow \mathbb{R}^{I_l}$ is the function whose *i*-th element is $\phi_i^{(l)}$ and $\mathbf{y}^{(l)} \in \mathbb{R}^{I_l}$ is the vector whose *i*-th element is $y_i^{(l)}$.

In each iteration of the L-BFGS algorithm, the image was updated by

$$\mathbf{v}_{t+1} = \mathbf{v}_t - \mathbf{H}_t \mathbf{g}_t \tag{4}$$

where $\mathbf{v}_t$ and $\mathbf{v}_{t+1}$ are the vectors before and after the *t*-th update, $\mathbf{g}_t$ is the gradient of the cost function at $\mathbf{v}_t$, and $\mathbf{H}_t$ is an approximation of the inverse hessian of the cost function at $\mathbf{v}_t$.

For each update, this gradient was calculated by the backpropagation algorithm as follows. Here, we define the backpropagated error $\delta_j^{(m)}$ as

$$\delta_j^{(m)} = \frac{\partial E}{\partial u_j^{(m)}} \tag{5}$$

where E is the cost function to be minimized and $u_j^{(m)}$ is the input to the j-th unit in the m-th layer in the forward path. Using the chain rule, $\delta_j^{(m)}$ can be calculated as a weighted sum of the backpropagated errors for the units in the (m + 1)-th layer:

$$\delta_j^{(m)} = f^{(m)\prime}\left(u_j^{(m)}\right) \sum_{k=1}^{I_{m+1}} w_{kj}^{(m+1)} \delta_k^{(m+1)} \tag{6}$$

where the function $f^{(m)\prime}$ is the derivative of the activation function between the m-th layer and (m + 1)-th layer. $w_{kj}^{(m+1)}$ is the weight between the j-th unit in the m-th layer and the k-th unit in the (m + 1)-th layer.

The backpropagated error for a unit in the last layer is given by

$$\delta_j^{(l)} = u_j^{(l)} - y_j^{(l)}, \tag{7}$$

and the gradient $\mathbf{g}_t$ is obtained as $\left(\delta_1^{(0)}, \delta_2^{(0)}, \cdots, \delta_{224\times224\times3}^{(0)}\right)^T$, which can be numerically calculated using the chain rule.

The calculation of the inverse hessian with a size of $I_l \times I_l$ is intractable because it requires huge memory. To avoid the memory problem, the inverse hessian was approximated based on the history of $\mathbf{g}_t$ and $\mathbf{v}_t$ following the update rule of the L-BFGS algorithm [8]. Each image was generated by 200 iterations and the spatially uniform image with the mean RGB contrast values of natural images was used as the initial image.

Also, the cost function was minimized by gradient descent with momentum [10]. In each iteration of the algorithm, the image was updated by

$$\mathbf{v}_{t+1} = \mathbf{v}_t + \boldsymbol{\mu}_t\ , \tag{8}$$

$$\boldsymbol{\mu}_{t+1} = m\boldsymbol{\mu}_t - \eta_t \mathbf{g}_t. \tag{9}$$

$\mathbf{v}_t$ and $\mathbf{v}_{t+1}$ are the vectors before and after the *t*-th update. $\mathbf{g}_t$ is the gradient of the cost function at $\mathbf{v}_t$, and $\boldsymbol{\mu}_t$ is a weighted average of the gradients from step 0 to *t*. The next update is determined based on the history of $\mathbf{g}_t$ to prevent $\mathbf{v}_t$ from oscillating around shallow local minima of the cost function. m is a parameter called the decay rate, and we set this to 0.9. η_t is the learning rate. Each image was generated by 200 iterations and η_t was linearly reduced from 2.0 to 0.0. The spatially uniform image with the mean RGB contrast values of natural images was used as the initial image for optimization.

For each update, the gradient $\mathbf{g}_t$ was calculated by the backpropagation algorithm with the procedure the same as for the L-BFGS algorithm.

Notes for supplementary movies. To reconstruct visual images, we first decoded (translated) measured brain activity patterns into deep neural network (DNN) features, and then fed these decoded features into a reconstruction algorithm. Our reconstruction algorithm starts from a given initial image and iteratively optimizes the pixel values so that the DNN features of the current image become similar to those decoded from brain activity. The videos can be viewed from our repository:
https://www.youtube.com/user/ATRDNI

References for Supporting Information

1. Engel SA, Rumelhart DE, Wandell BA, Lee AT, Glover GH, Chichilnisky E, et al. fMRI of human visual cortex. *Nature*. 1994; 369: 525. doi: 10.1038/369525a0.
2. Sereno MI, Dale AM, Reppas JB, Kwong KK, Belliveau JW, Brady TJ, et al. Borders of multiple visual areas in humans revealed by functional magnetic resonance imaging. *Science*. 1995; 268: 889–893. doi: 10.1126/science.7754376.
3. Kourtzi Z, Kanwisher N. Cortical regions involved in perceiving object shape. *J Neurosci*. 2000; 20: 3310–3318. doi: 10.1523/JNEUROSCI.20-09-03310.2000.
4. Kanwisher N, McDermott J, Chun MM. The fusiform face area: a module in human extrastriate cortex specialized for face perception. *J Neurosci.* 1997; 17: 4302–4311. doi: 10.1523/JNEUROSCI.17-11-04302.1997.
5. Epstein R, Kanwisher N. A cortical representation of the local visual environment. *Nature*. 1998; 392: 598–601. doi: 10.1038/33402.
6. Deng J, Dong W, Socher R, Li L, Li K, Fei-Fei L. Imagenet: A large-scale hierarchical image database. *Proc IEEE Comput Soc Conf Comput Vis Pattern Recognit.* 2009; 248–255. doi: 10.1109/CVPR.2009.5206848.
7. Le QV, Ngiam J, Coates A, Lahiri A, Prochnow B, Ng AY. On optimization methods for deep learning. *Proc Int Conf Mach Learn* (Bellevue, Washington, USA), 2011; 265–272.
8. Liu DC, Nocedal J. On the limited memory BFGS method for large scale optimization. *Math Program*. 1989; 45: 503–528. doi: 10.1007/BF01589116.
9. Gatys LA, Ecker AS, Bethge M. Image style transfer using convolutional neural networks. *Proc IEEE Comput Soc Conf Comput Vis Pattern Recognit.* 2016; 2414–2423. doi: 10.1109/CVPR.2016.265.
10. Qian N. On the momentum term in gradient descent learning algorithms. *Neural Netw.* 1999; 12: 145–151. doi: 10.1016/S0893-6080(98)00116-6.
11. Mahendran A, Vedaldi A. Understanding deep image representations by inverting them. *Proc IEEE Comput Soc Conf Comput Vis Pattern Recognit*. 2015; 5188–5196. doi: 10.1109/CVPR.2015.7299155.

12. Horikawa T, Kamitani Y. Generic decoding of seen and imagined objects using hierarchical visual features. *Nat Commun*. 2017; 8: 15037. doi: 10.1038/ncomms15037.
