# Supplementary material for: Deep image reconstruction from human brain activity
Source: PLoS Comput Biol. 2019 Jan 14;15(1):e1006633. doi: 10.1371/journal.pcbi.1006633 (PMC6347330; doi:10.1371/journal.pcbi.1006633)
Supplement: S2 Fig — The black and gray surrounding frames indicate presented and reconstructed images respectively (VC activity, DNN 1–8, with the DGN). The three columns of reconstructed images correspond to reconstructions from three subjects. For copyright reasons, we present only a subset of the 50 test natural images; those for which the copyright holders gave us permission to use. (PDF) [file pcbi.1006633.s003.pdf]

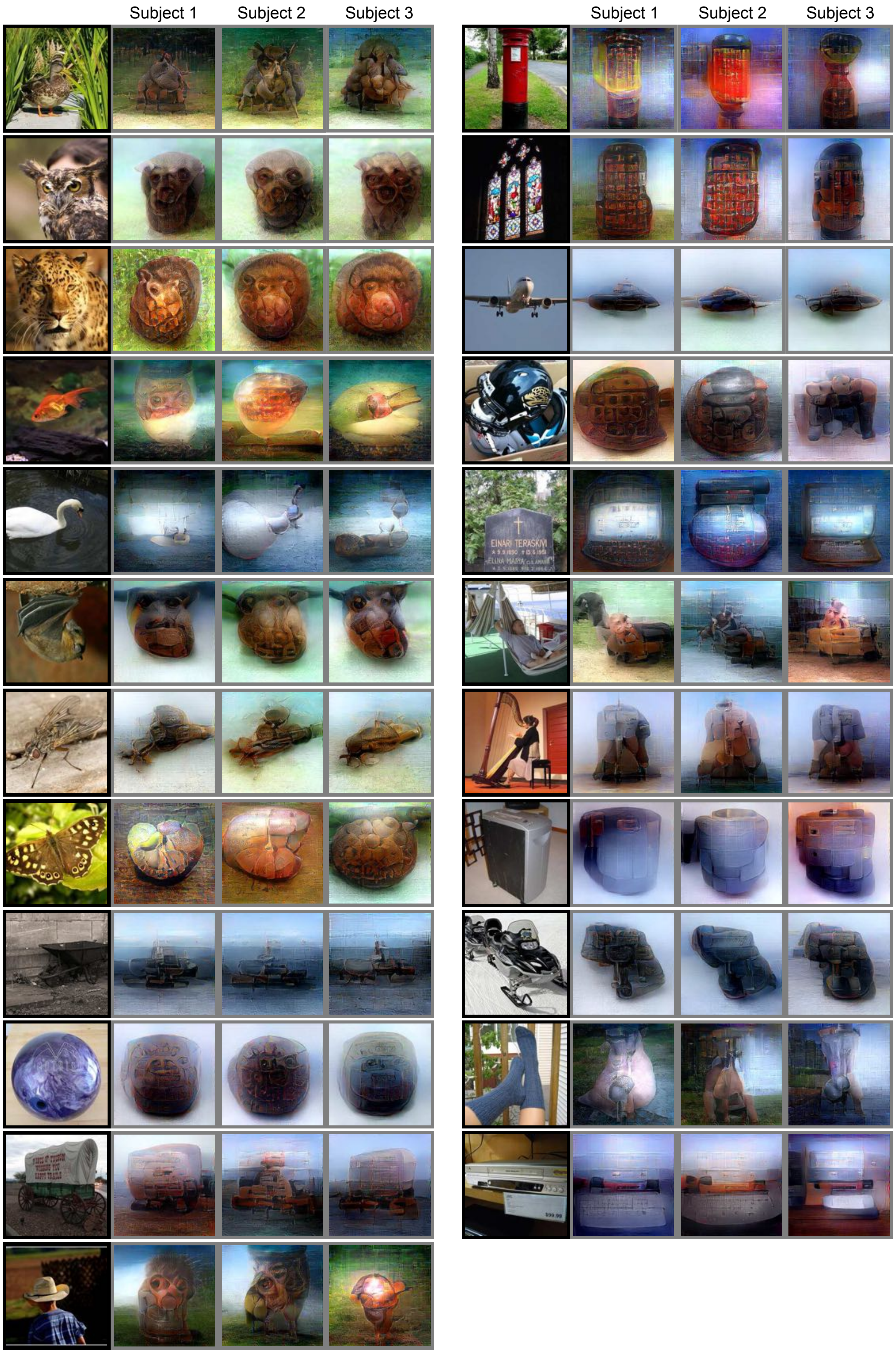
Subject 1
Subject 2
Subject 3
Subject 1
Subject 2
Subject 3

S2 Fig. Examples of natural image reconstructions obtained with the DGN. The black and gray surrounding frames indicate presented and reconstructed images respectively (VC activity, DNN 1–8, with the DGN). The three columns of reconstructed images correspond to reconstructions from three subjects. For copyright reasons, we present only a subset of the 50 test natural images; those for which the copyright holders gave us permission to use.
