# Supplementary material for: Deep image reconstruction from human brain activity
Source: PLoS Comput Biol. 2019 Jan 14;15(1):e1006633. doi: 10.1371/journal.pcbi.1006633 (PMC6347330; doi:10.1371/journal.pcbi.1006633)
Supplement: S3 Fig — The same reconstruction analysis was performed with a previously published dataset [10] (VC activity, DNN 1–8, with the DGN). See Horikawa & Kamitani (2017) [10] for details of the data. The black and gray surrounding frames indicate presented and reconstructed images respectively. The five columns of reconstructed images correspond to reconstructions from five subjects. (PDF) [file pcbi.1006633.s004.pdf]

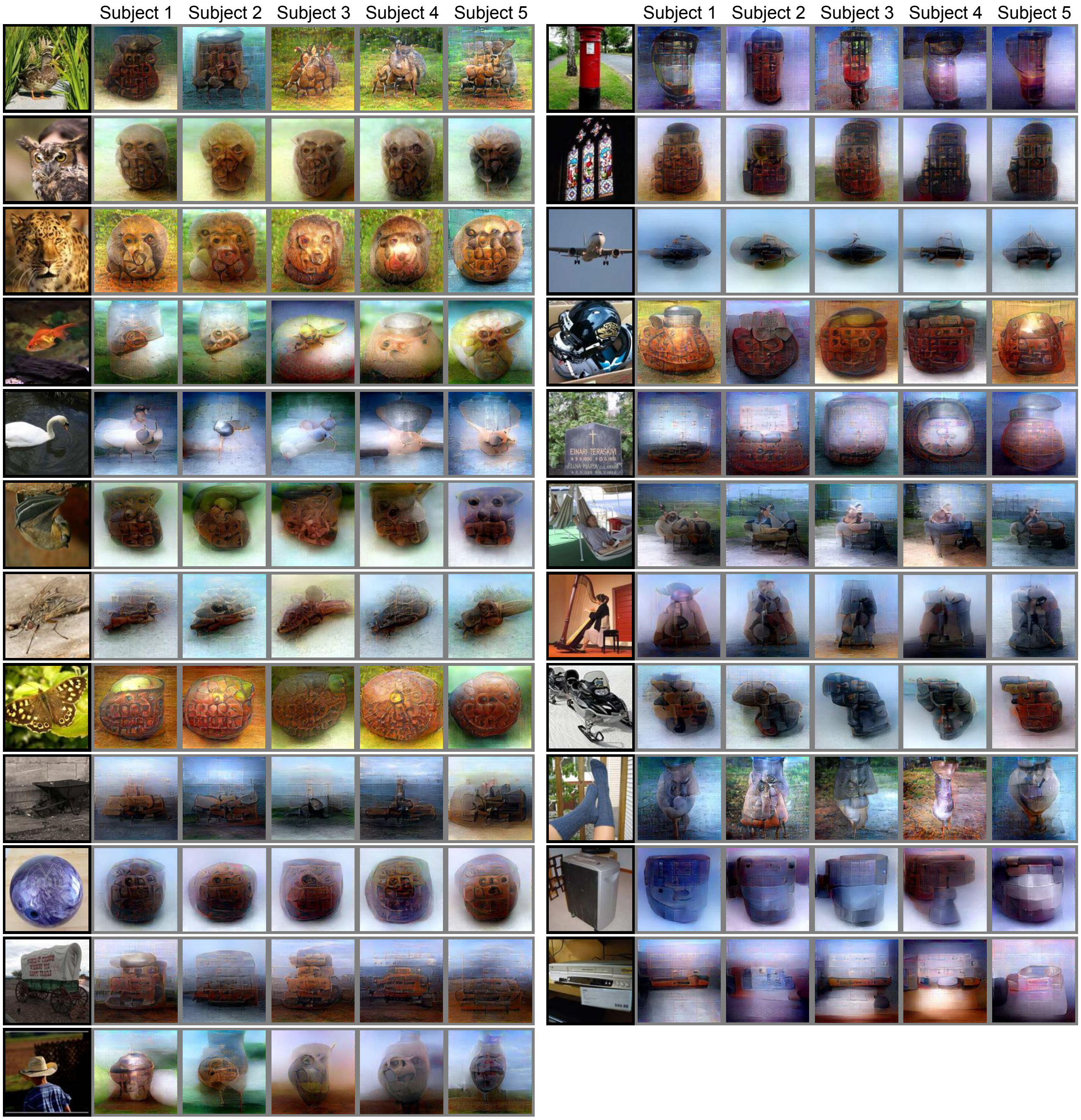

S3 Fig. Reconstructions from the generic object decoding dataset. The same reconstruction analysis was performed with a previously published dataset [12] (VC activity, DNN 1–8, with the DGN). See Horikawa & Kamitani (2017) [12] for details of the data. The black and gray surrounding frames indicate presented and reconstructed images respectively. The five columns of reconstructed images correspond to reconstructions from five subjects.
