# Supplementary material for: Deep image reconstruction from human brain activity
Source: PLoS Comput Biol. 2019 Jan 14;15(1):e1006633. doi: 10.1371/journal.pcbi.1006633 (PMC6347330; doi:10.1371/journal.pcbi.1006633)
Supplement: S4 Fig — Evaluations on individual subjects’ results are separately shown (VC activity; DNN1–8; N = 50; chance level, 50%; cf., Fig 3B), indicating that overall tendency was almost consistent across different subjects, except that the human judgment accuracy of reconstructions from Subject 3 showed slightly higher accuracy without the DGN than that with the DGN. Evaluations of reconstructions using pixel-wise spatial correlation for Subject 1–3 showed 78.4%, 74.2%, and 75.7% with the DGN, and 80.4%, 77.2%, and 81.3% without the DGN, respectively. Evaluations of reconstructions using human judgment for Subject 1–3 showed 98.5%, 97.3%, and 95.3% with the DGN, and 96.6%, 94.7%, and 96.7% without the DGN, respectively. (PDF) [file pcbi.1006633.s005.pdf]

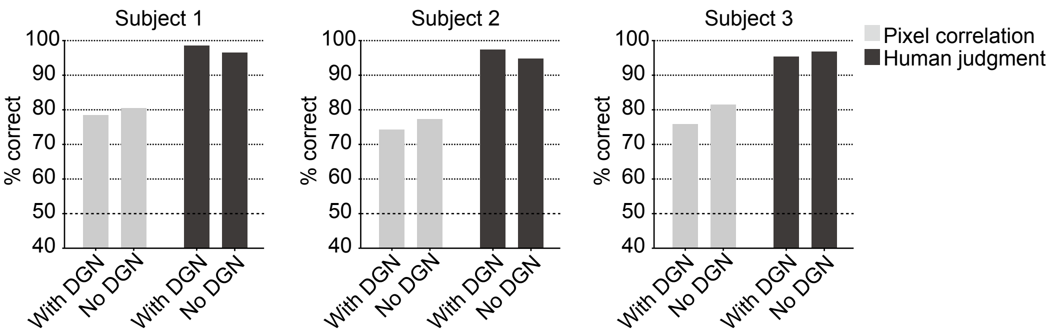

S4 Fig. Reconstruction quality of seen natural images for individual subjects. Evaluations on individual subjects' results are separately shown (VC activity; DNN1–8; $N = 50$; chance level, 50%; cf., Fig 3B), indicating that overall tendency was almost consistent across different subjects, except that the human judgment accuracy of reconstructions from Subject 3 showed slightly higher accuracy without the DGN than that with the DGN. Evaluations of reconstructions using pixel-wise spatial correlation for Subject 1–3 showed 78.4%, 74.2%, and 75.7% with the DGN, and 80.4%, 77.2%, and 81.3% without the DGN, respectively. Evaluations of reconstructions using human judgment for Subject 1–3 showed 98.5%, 97.3%, and 95.3% with the DGN, and 96.6%, 94.7%, and 96.7% without the DGN, respectively.
