# Supplementary material for: Deep image reconstruction from human brain activity
Source: PLoS Comput Biol. 2019 Jan 14;15(1):e1006633. doi: 10.1371/journal.pcbi.1006633 (PMC6347330; doi:10.1371/journal.pcbi.1006633)
Supplement: S5 Fig — The black and gray surrounding frames indicate presented and reconstructed images respectively (VC activity, DNN 1–8, without the DGN). The three columns of reconstructed images correspond to reconstructions from three subjects. (PDF) [file pcbi.1006633.s006.pdf]

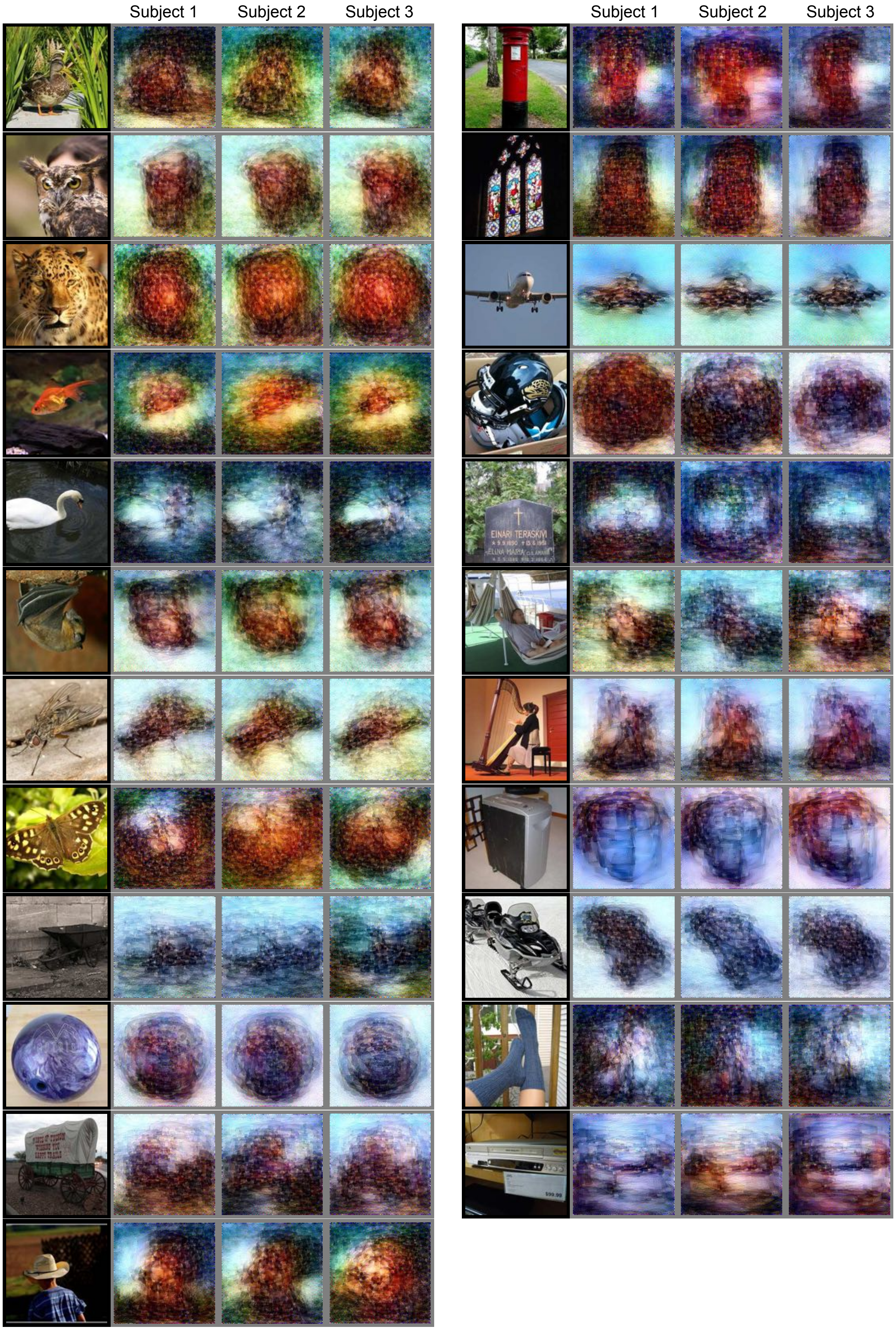
Subject 1
Subject 2
Subject 3
Subject 1
Subject 2
Subject 3

S5 Fig. Other examples of natural image reconstructions obtained without the DGN. The black and gray surrounding frames indicate presented and reconstructed images respectively (VC activity, DNN 1–8, without the DGN). The three columns of reconstructed images correspond to reconstructions from three subjects.
