# Supplementary material for: Deep image reconstruction from human brain activity
Source: PLoS Comput Biol. 2019 Jan 14;15(1):e1006633. doi: 10.1371/journal.pcbi.1006633 (PMC6347330; doi:10.1371/journal.pcbi.1006633)
Supplement: S6 Fig — The black and gray surrounding frames indicate presented and reconstructed images respectively (VC activity, DNN 1–8). We used different initial states for reconstructions with and without the DGN. For reconstructions with the DGN, we additionally performed the reconstruction analysis using a Gaussian-random-value vector (mean = 0, standard deviation = 1) as the initial state as well as the zero-value vector (main analysis; e.g., Fig 2). For reconstructions without the DGN, we also performed reconstructions from a uniform-random-value image (ranged between 0 and 255) and the zero-value image in addition to the spatially uniform image with the mean RGB values of natural images (main analysis; e.g., Fig 3). For comparison, reconstructed images from different initial states are compared within the same subjects. The results showed slightly different but almost equivalent images from different initial states, demonstrating the stability of our reconstructions. (PDF) [file pcbi.1006633.s007.pdf]

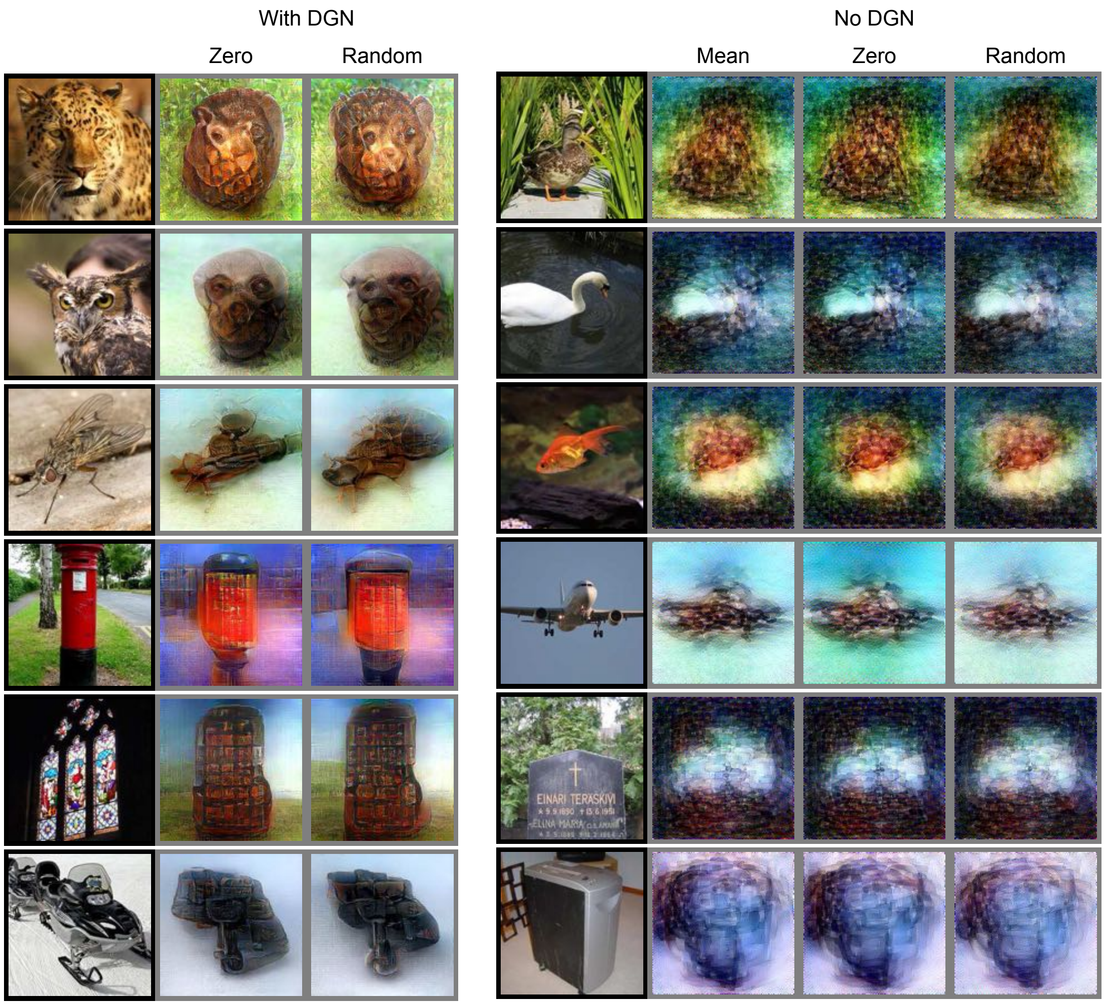

S6 Fig. Reconstructions from different initial states. The black and gray surrounding frames indicate presented and reconstructed images respectively (VC activity, DNN 1–8). We used different initial states for reconstructions with and without the DGN. For reconstructions with the DGN, we additionally performed the reconstruction analysis using a Gaussian-random-value vector (mean = 0, standard deviation = 1) as the initial state as well as the zero-value vector (main analysis; e.g., Fig 2). For reconstructions without the DGN, we also performed reconstructions from a uniform-random-value image (ranged between 0 and 255) and the zero-value image in addition to the spatially uniform image with the mean RGB values of natural images (main analysis; e.g., Fig 3). For comparison, reconstructed images from different initial states are compared within the same subjects. The results showed slightly different but almost equivalent images from different initial states, demonstrating the stability of our reconstructions.
