# Supplementary material for: Deep image reconstruction from human brain activity
Source: PLoS Comput Biol. 2019 Jan 14;15(1):e1006633. doi: 10.1371/journal.pcbi.1006633 (PMC6347330; doi:10.1371/journal.pcbi.1006633)
Supplement: S7 Fig — The black and gray surrounding frames indicate presented and reconstructed images respectively (VC activity, without the DGN). (PDF) [file pcbi.1006633.s008.pdf]

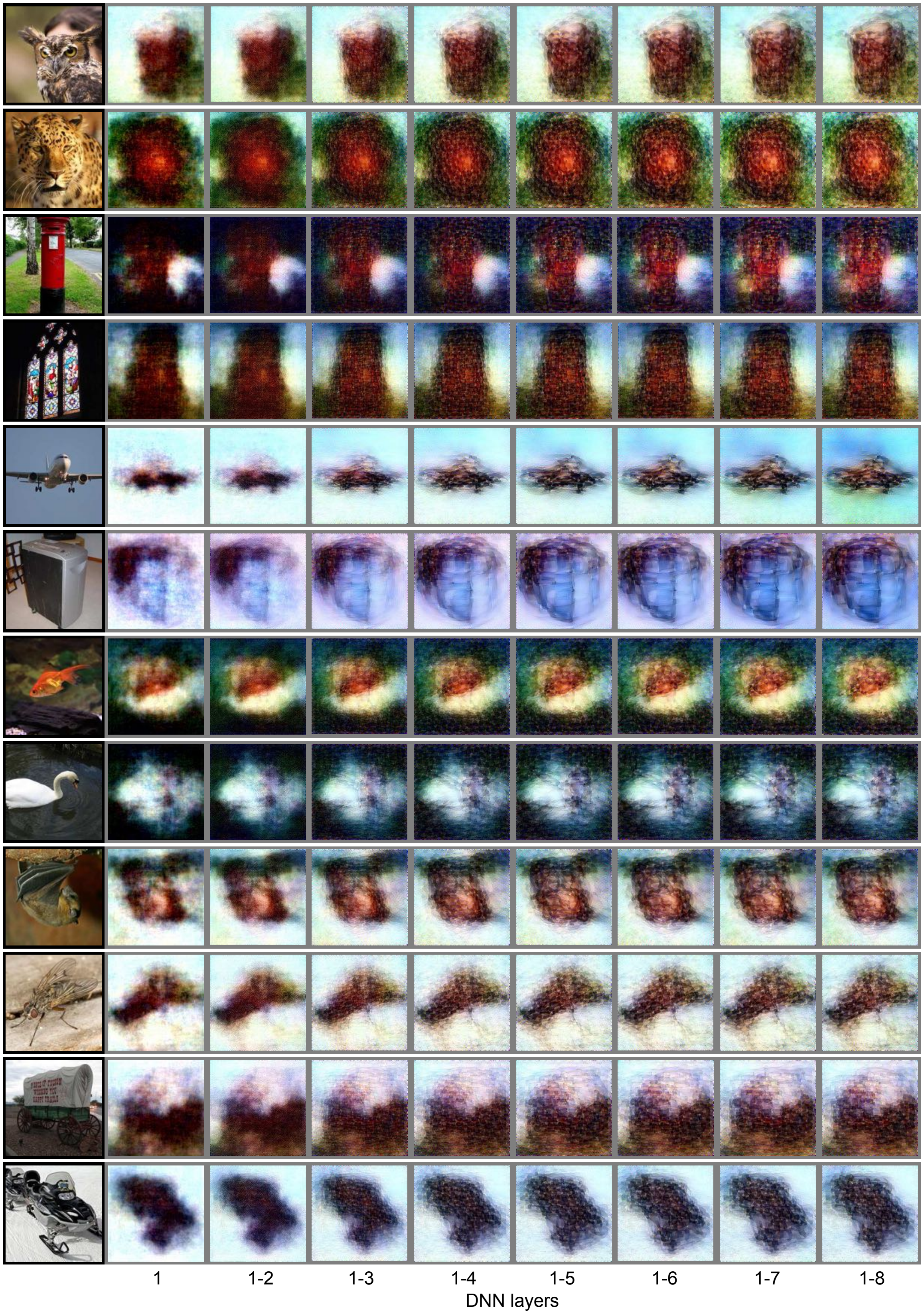
1
1-2
1-3
1-4
1-5
1-6
1-7
1-8
DNN layers

S7 Fig. Other examples of reconstructions with a variable number of multiple DNN layers. The black and gray surrounding frames indicate presented and reconstructed images respectively (VC activity, without the DGN).
