# Supplementary material for: Deep image reconstruction from human brain activity
Source: PLoS Comput Biol. 2019 Jan 14;15(1):e1006633. doi: 10.1371/journal.pcbi.1006633 (PMC6347330; doi:10.1371/journal.pcbi.1006633)
Supplement: S8 Fig — The black and gray surrounding frames indicate presented and reconstructed images respectively (without the DGN). We used DNN features from individual layers (DNN1, DNN2, …, or DNN8) as well as the combination of all DNN layers (DNN1–8) for the reconstruction analysis, in which either of true or decoded features (VC activity) were provided to the reconstruction algorithm. While reconstructions from individual layers, especially from higher layers, showed poorer reconstruction quality even from true features, combining multiple layers produced almost complete reconstructions of original images from true features and good reconstructions from decoded features (cf., Fig 4 and S7 Fig). (PDF) [file pcbi.1006633.s009.pdf]

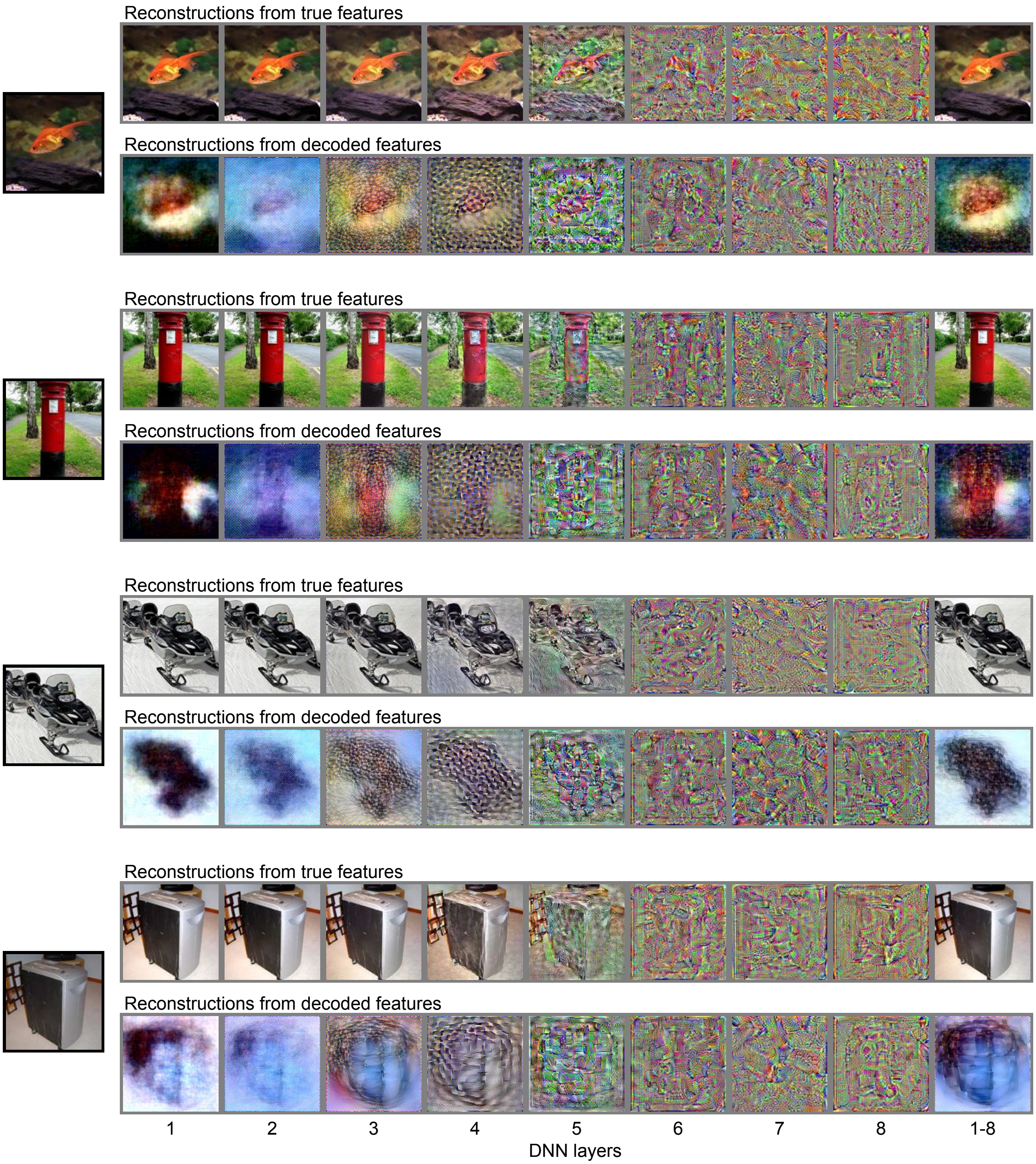

S8 Fig. Examples of reconstructions from individual DNN layers. The black and gray surrounding frames indicate presented and reconstructed images respectively (without the DGN). We used DNN features from individual layers (DNN1, DNN2, …, or DNN8) as well as the combination of all DNN layers (DNN1–8) for the reconstruction analysis, in which either of true or decoded features (VC activity) were provided to the reconstruction algorithm. While reconstructions from individual layers, especially from higher layers, showed poorer reconstruction quality even from true features, combining multiple layers

produced almost complete reconstructions of original images from true features and good reconstructions from decoded features (cf., Fig 4 and S7 Fig).
