# Supplementary material for: Deep image reconstruction from human brain activity
Source: PLoS Comput Biol. 2019 Jan 14;15(1):e1006633. doi: 10.1371/journal.pcbi.1006633 (PMC6347330; doi:10.1371/journal.pcbi.1006633)
Supplement: S9 Fig — To investigate the relations between feature decoding accuracy and reconstruction quality, we first evaluated feature decoding accuracies for individual samples instead of those for individual DNN units (cf., S1 Fig; see Materials and Methods: “Evaluation of reconstruction quality” for how to evaluate the reconstruction quality for individual samples). To evaluate the feature decoding accuracy for each sample, Pearson’s correlation coefficients were caluculated between the decoded and true feature values for a single stimulus image using all units within each layer. To avoid estimating spuriously high correlations due to baseline and scale differences across units, feature values of each unit of the test data (test natural-image) were z-normalized using means and standard deviations estimated from the training data (training natural-image) before calculating correlations. Using the estimated feature decoding accuracy and reconstruction quality for individual samples (N = 50), Pearson’s correlation coefficients were further calculated between the reconstruction quality (VC activity; with or without the DGN, DNN1–8) and the feature decoding accuracy from individual layers or mean accuracy averaged across 19 layers. While the correlations varied across layers and subjects, the results on average showed positive correlations between the feature decoding accuracy and the reconstruction quality for all combinations of the assessments and the reconstruction algorithms, suggesting that higher decoding accuracy would lead to better reconstruction quality. Interestingly, the analysis showed distinct correlation patterns across layers between the two assessment types, showing that high correlaions were specifically observed from early layers with the pixel-wise spatial correlations although moderately high correlations were observed rather evenly from most layers with the human judgment. These results may reflect the different characteristics of the two assessments, indicating [file pcbi.1006633.s010.pdf]

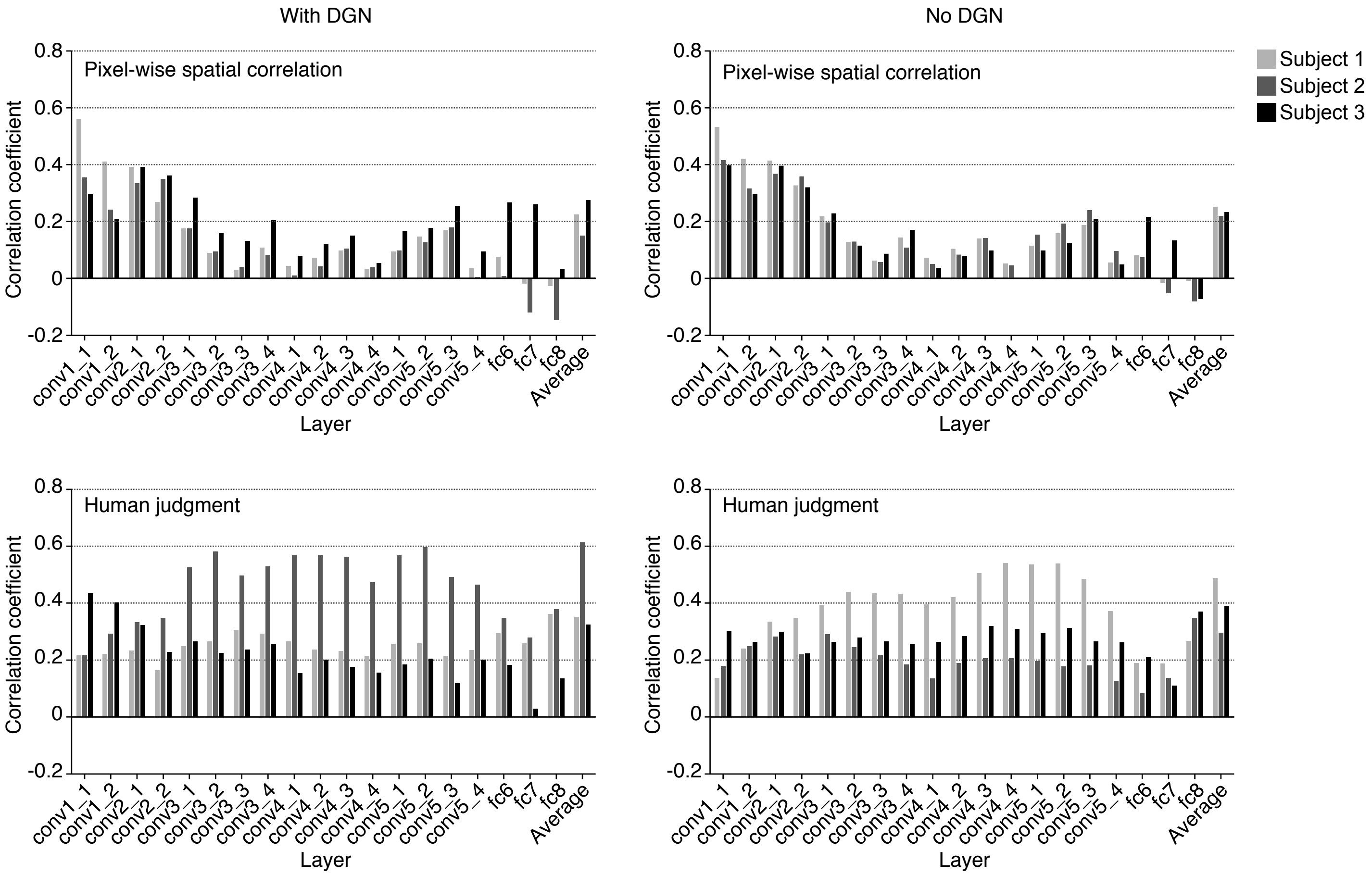

S9 Fig. Correlations between feature decoding accuracy and reconstruction quality. To investigate the relations between feature decoding accuracy and reconstruction quality, we first evaluated feature decoding accuracies for individual samples instead of those for individual DNN units (cf., S1 Fig; see Materials and Methods: "Evaluation of reconstruction quality" for how to evaluate the reconstruction quality for individual samples). To evaluate the feature decoding accuracy for each sample, Pearson's correlation coefficients were caluculated between the decoded and true feature values for a single stimulus image using all units within each layer. To avoid estimating spuriously high correlations due to baseline and scale differences across units, feature values of each unit of the test data (test natural-image) were z-normalized using means and standard deviations estimated from the training data (training natural-image) before calculating correlations. Using the estimated feature decoding accuracy and reconstruction quality for individual samples ($N = 50$), Pearson's correlation coefficients were further calculated between the reconstruction quality (VC activity; with or without the DGN, DNN1–8) and the feature decoding accuracy from individual layers or mean accuracy averaged across 19 layers. While the correlations varied across layers and subjects, the results on average showed positive correlations between the feature decoding accuracy and the

reconstruction quality for all combinations of the assessments and the reconstruction algorithms, suggesting that higher decoding accuracy would lead to better reconstruction quality. Interestingly, the analysis showed distinct correlation patterns across layers between the two assessment types, showing that high correlaions were specifically observed from early layers with the pixel-wise spatial correlations although moderately high correlations were observed rather evenly from most layers with the human judgment. These results may reflect the different characteristics of the two assessments, indicating that the pixel-wise correlation is suited to evaluate accuracy in low-level features whereas the human judgment is capable of evaluating accuracy in multiple-levels of visual features.
