# Supplementary material for: Deep image reconstruction from human brain activity
Source: PLoS Comput Biol. 2019 Jan 14;15(1):e1006633. doi: 10.1371/journal.pcbi.1006633 (PMC6347330; doi:10.1371/journal.pcbi.1006633)
Supplement: S10 Fig — The black and gray surrounding frames indicate presented and reconstructed images respectively (VC activity, DNN 1–8, without the DGN). The three rows of reconstructed images correspond to reconstructions from three subjects. (PDF) [file pcbi.1006633.s011.pdf]

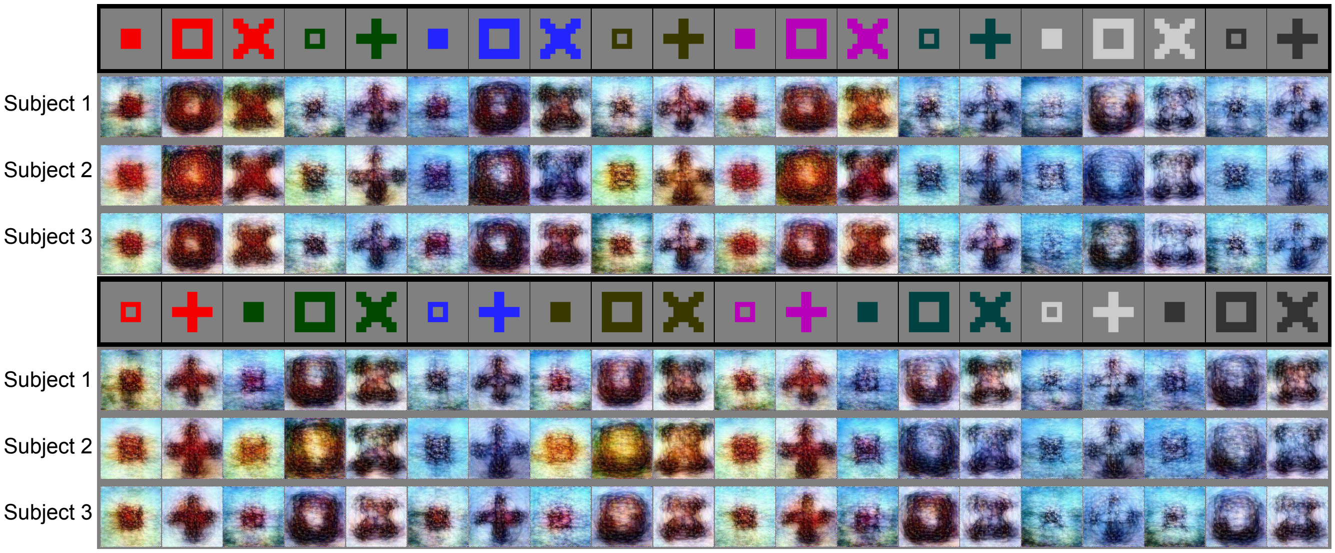

S10 Fig. All examples of artificial shape reconstructions. The black and gray surrounding frames indicate presented and reconstructed images respectively (VC activity, DNN 1–8, without the DGN). The three rows of reconstructed images correspond to reconstructions from three subjects.
