# Supplementary material for: Deep image reconstruction from human brain activity
Source: PLoS Comput Biol. 2019 Jan 14;15(1):e1006633. doi: 10.1371/journal.pcbi.1006633 (PMC6347330; doi:10.1371/journal.pcbi.1006633)
Supplement: S11 Fig — The black and gray surrounding frames indicate presented and reconstructed images respectively (VC activity, DNN 1–8, without the DGN). The three rows of reconstructed images correspond to reconstructions from three subjects. (PDF) [file pcbi.1006633.s012.pdf]

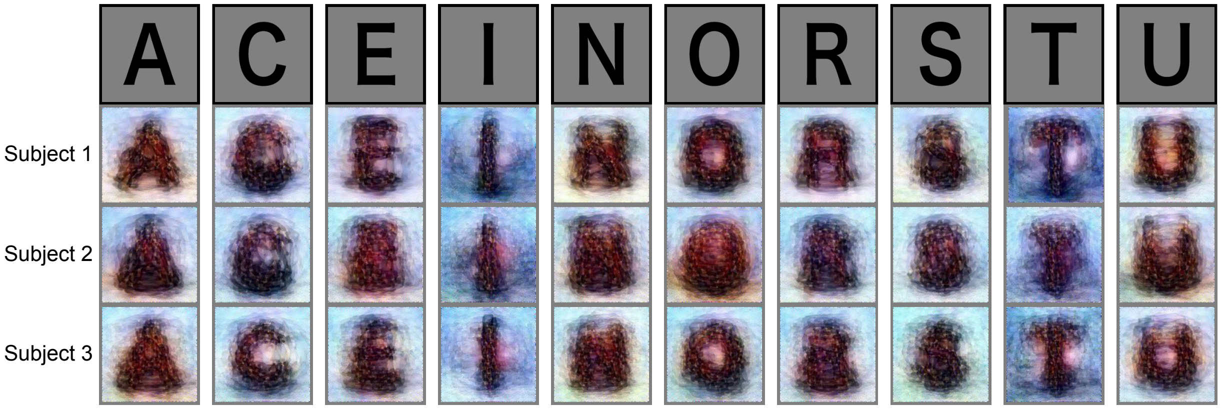

S11 Fig. All examples of alphabetical letter reconstructions. The black and gray surrounding frames indicate presented and reconstructed images respectively (VC activity, DNN 1–8, without the DGN). The three rows of reconstructed images correspond to reconstructions from three subjects.
