# Supplementary material for: Deep image reconstruction from human brain activity
Source: PLoS Comput Biol. 2019 Jan 14;15(1):e1006633. doi: 10.1371/journal.pcbi.1006633 (PMC6347330; doi:10.1371/journal.pcbi.1006633)
Supplement: S12 Fig — Evaluations on individual subjects’ results are separately shown (VC activity; DNN1–8; without the DGN; N = 40; chance level, 50%; cf., Fig 6C left). Evaluations of reconstructions using pixel-wise spatial correlation showed 69.6%, 72.1%, and 69.8% for Subject 1–3, respectively. Evaluations of reconstructions using human judgment showed 91.7%, 91.3%, and 90.1% for Subject 1–3, respectively. (PDF) [file pcbi.1006633.s013.pdf]

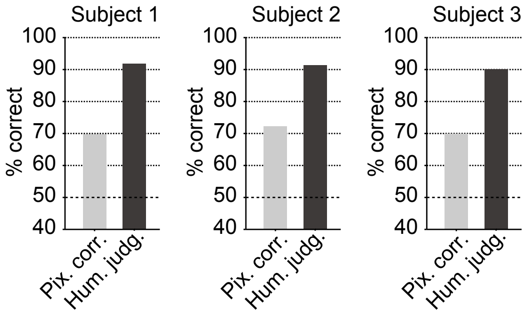

S12 Fig. Reconstruction quality of artificial shapes for individual subjects.
Evaluations on individual subjects' results are separately shown (VC activity; DNN1–8; without the DGN; $N = 40$; chance level, 50%; cf., Fig 6C left). Evaluations of reconstructions using pixel-wise spatial correlation showed 69.6%, 72.1%, and 69.8% for Subject 1–3, respectively. Evaluations of reconstructions using human judgment showed 91.7%, 91.3%, and 90.1% for Subject 1–3, respectively.
