# Supplementary material for: Deep image reconstruction from human brain activity
Source: PLoS Comput Biol. 2019 Jan 14;15(1):e1006633. doi: 10.1371/journal.pcbi.1006633 (PMC6347330; doi:10.1371/journal.pcbi.1006633)
Supplement: S13 Fig — Evaluations on individual subjects’ results are separately shown (VC activity; DNN1–8; without the DGN; N = 10; chance level, 50%; cf., Fig 6C right). Evaluations of reconstructions using pixel-wise spatial correlation showed 98.9%, 87.8%, and 100.0% for Subject 1–3, respectively. Evaluations of reconstructions using human judgment showed 100.0%, 98.9%, and 100.0% for Subject 1–3, respectively. (PDF) [file pcbi.1006633.s014.pdf]

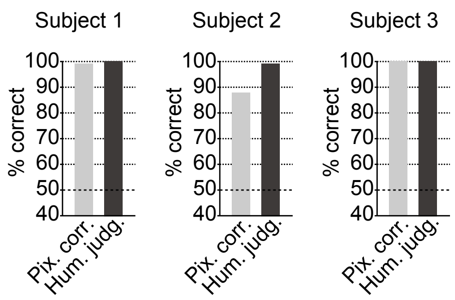

S13 Fig. Reconstruction quality of alphabetical letters for individual subjects. Evaluations on individual subjects' results are separately shown (VC activity; DNN1–8; without the DGN; $N = 10$; chance level, 50%; cf., Fig 6C right). Evaluations of reconstructions using pixel-wise spatial correlation showed 98.9%, 87.8%, and 100.0% for Subject 1–3, respectively. Evaluations of reconstructions using human judgment showed 100.0%, 98.9%, and 100.0% for Subject 1–3, respectively.
