# Supplementary material for: Deep image reconstruction from human brain activity
Source: PLoS Comput Biol. 2019 Jan 14;15(1):e1006633. doi: 10.1371/journal.pcbi.1006633 (PMC6347330; doi:10.1371/journal.pcbi.1006633)
Supplement: S14 Fig — The black and gray surrounding frames indicate presented and reconstructed images respectively (DNN 1–8, without the DGN). (PDF) [file pcbi.1006633.s015.pdf]

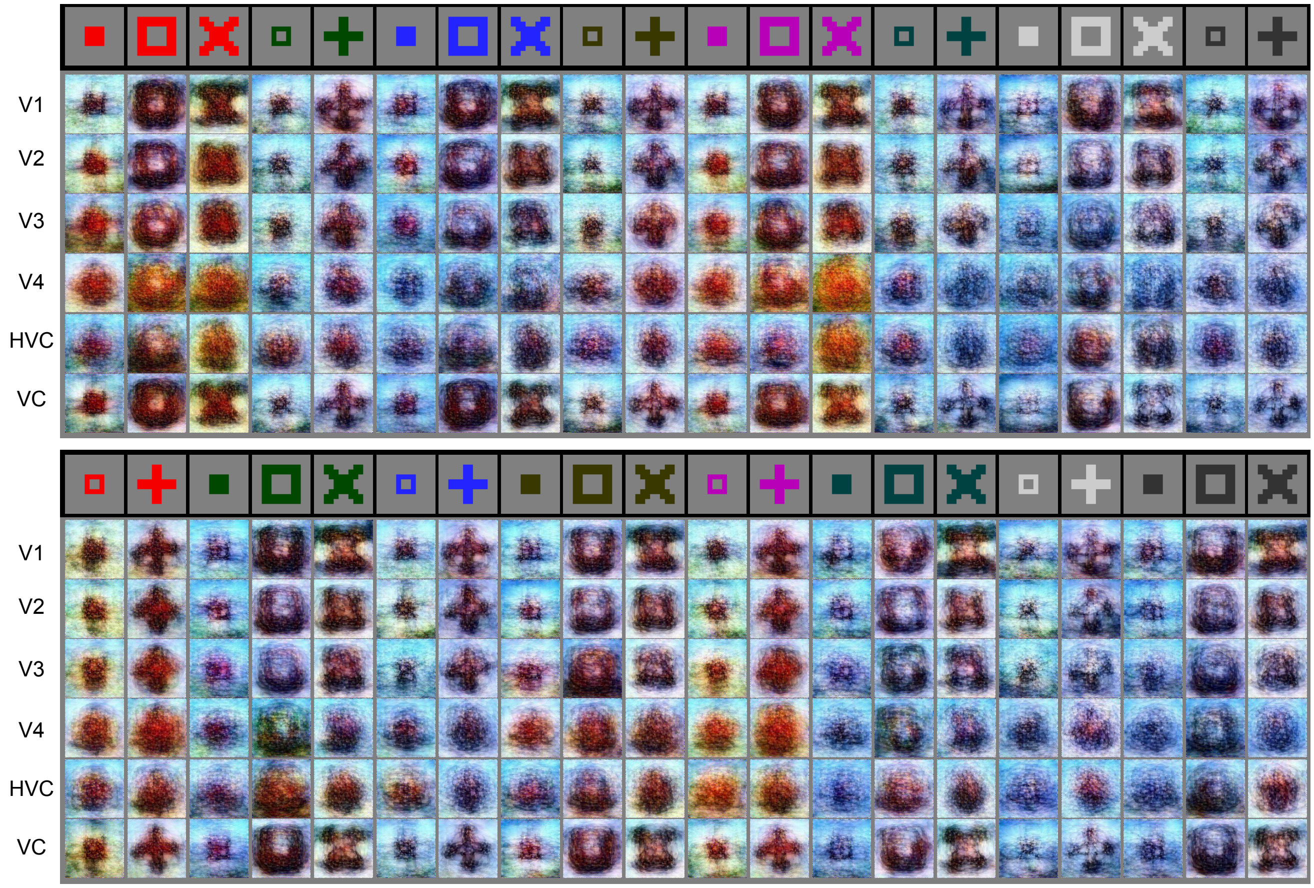

S14 Fig. All examples of artificial shape reconstructions obtained from different visual areas (Subject 1). The black and gray surrounding frames indicate presented and reconstructed images respectively (DNN 1–8, without the DGN).
