# Supplementary material for: Deep image reconstruction from human brain activity
Source: PLoS Comput Biol. 2019 Jan 14;15(1):e1006633. doi: 10.1371/journal.pcbi.1006633 (PMC6347330; doi:10.1371/journal.pcbi.1006633)
Supplement: S15 Fig — Evaluations on individual subjects’ results are separately shown (DNN1–8; without the DGN; N = 40; chance level, 50%; cf., Fig 7B). Evaluations by pixel-wise correlations and human judgment both showed almost consistent tendency across different subjects, showing that shapes were reconstructed better from early visual areas, whereas colors were reconstructed better from relatively higher visual areas. (PDF) [file pcbi.1006633.s016.pdf]

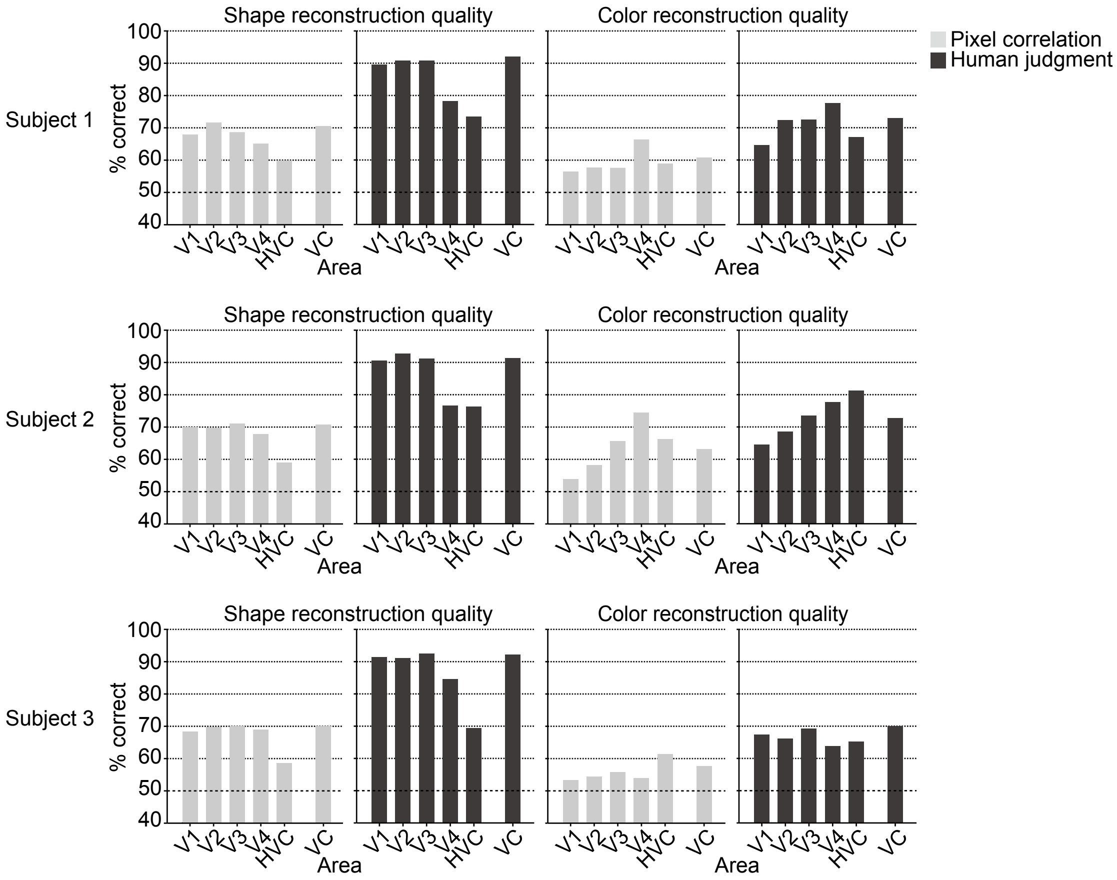

S15 Fig. Reconstruction quality of shape and color for different visual areas for individual subjects. Evaluations on individual subjects' results are separately shown (DNN1–8; without the DGN; $N = 40$; chance level, 50%; cf., Fig 7B). Evaluations by pixel-wise correlations and human judgment both showed almost consistent tendency across different subjects, showing that shapes were reconstructed better from early visual areas, whereas colors were reconstructed better from relatively higher visual areas.
