# Supplementary material for: Deep image reconstruction from human brain activity
Source: PLoS Comput Biol. 2019 Jan 14;15(1):e1006633. doi: 10.1371/journal.pcbi.1006633 (PMC6347330; doi:10.1371/journal.pcbi.1006633)
Supplement: S16 Fig — The black and gray surrounding frames indicate presented and reconstructed images respectively (VC activity, DNN 1–8, without the DGN). The three rows of reconstructed images correspond to reconstructions from three subjects. The rightmost images in the bottom row show reconstructions during maintenance of fixation without imagery. (PDF) [file pcbi.1006633.s017.pdf]

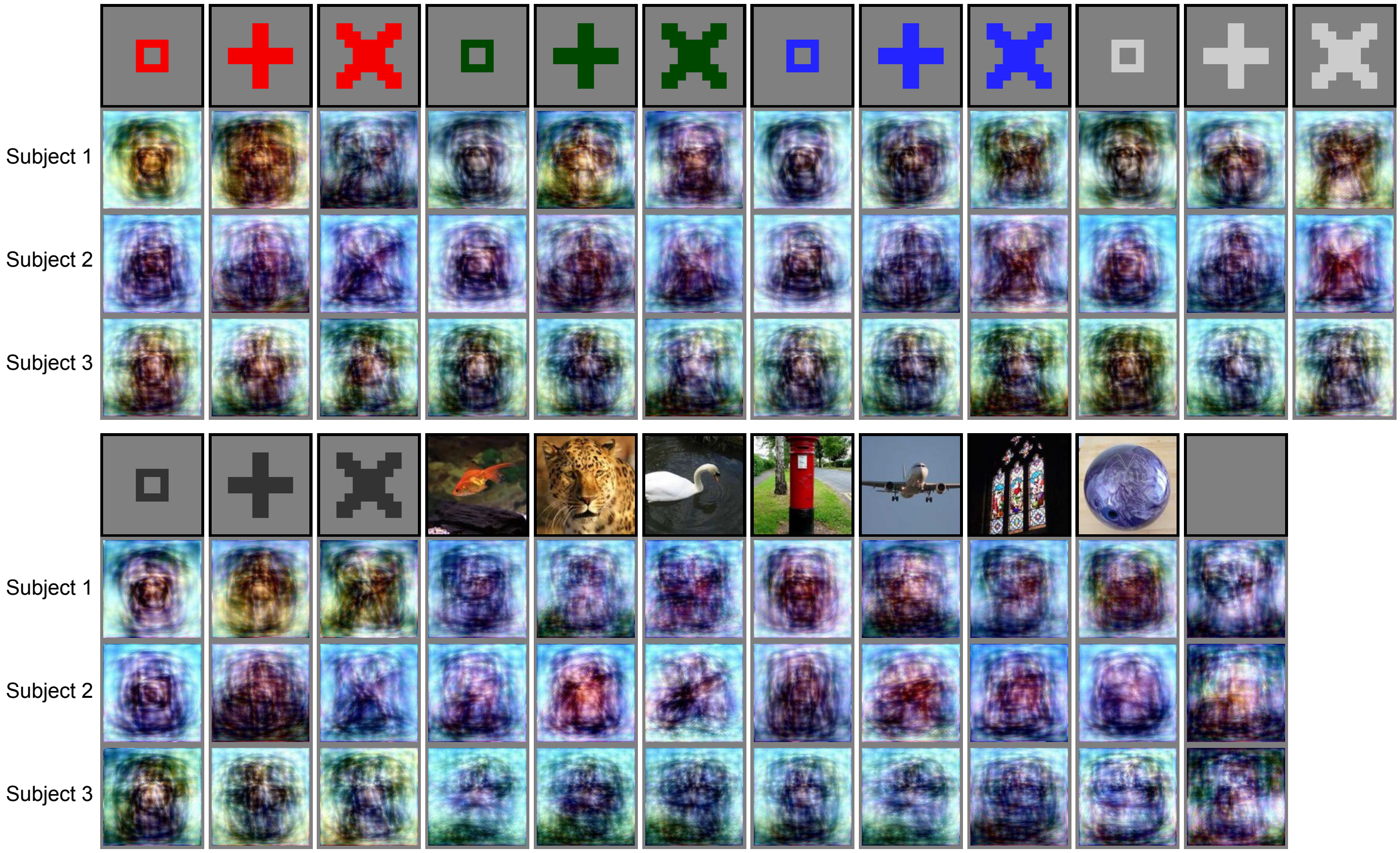

S16 Fig. Other examples of imagery image reconstructions. The black and gray surrounding frames indicate presented and reconstructed images respectively (VC activity, DNN 1–8, without the DGN). The three rows of reconstructed images correspond to reconstructions from three subjects. The rightmost images in the bottom row show reconstructions during maintenance of fixation without imagery.
