# Supplementary material for: Deep image reconstruction from human brain activity
Source: PLoS Comput Biol. 2019 Jan 14;15(1):e1006633. doi: 10.1371/journal.pcbi.1006633 (PMC6347330; doi:10.1371/journal.pcbi.1006633)
Supplement: S17 Fig — Vividness scores reported during the imagery experiment are shown in descending order of mean vividness scores across trials for individual images. For each subject, the vividness scores were averaged across trials for the same imagery images (N = 20). For the pooled results, to eliminate baseline and variability differences across subjects, the vividness scores obtained from individual subjects were first converted to z-scores within each subject, and then averaged across all trials from three subjects (N = 60). The rightmost two bars indicated as “artificial” and “natural” show mean vividness scores separately pooled for artificial shapes (15 artificial shapes) and natural images (10 natural images). Error bars indicate 95% confidence intervals across trials. (PDF) [file pcbi.1006633.s018.pdf]

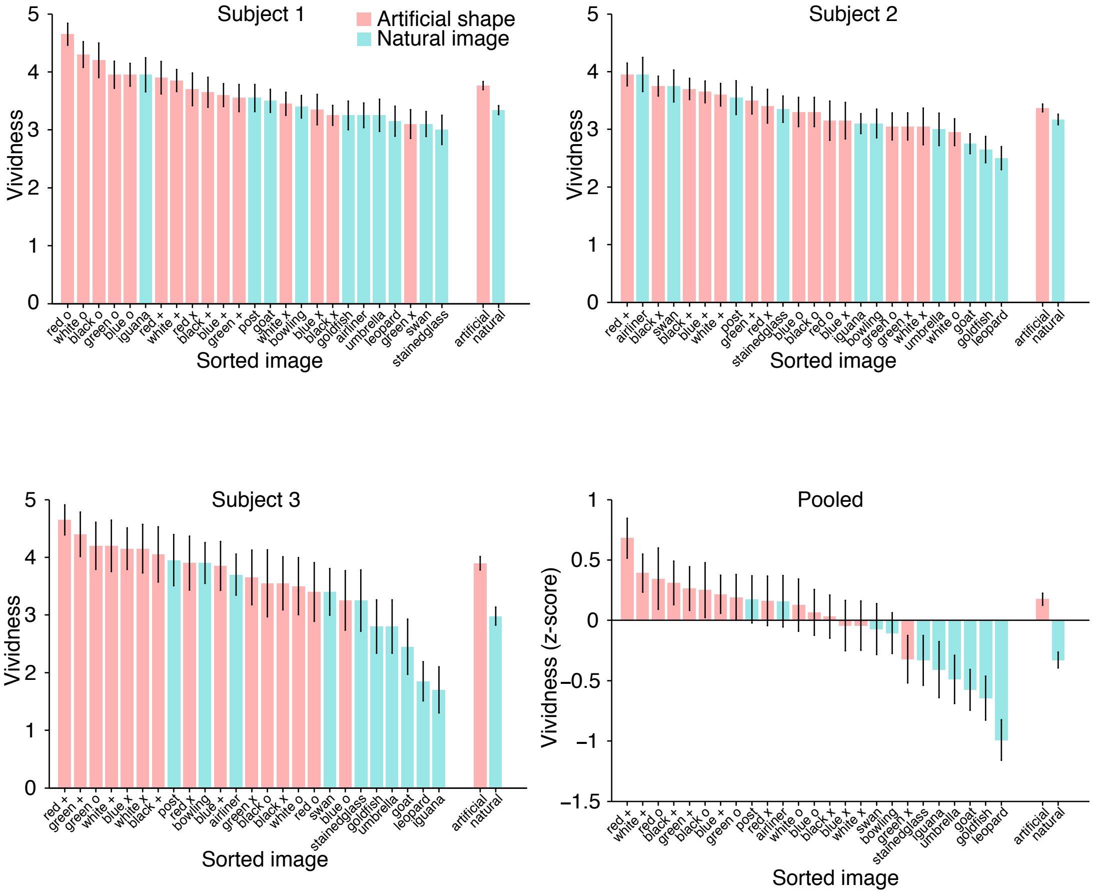

S17 Fig. Vividness scores for imagery images reported by subjects. Vividness scores reported during the imagery experiment are shown in descending order of mean vividness scores across trials for individual images. For each subject, the vividness scores were averaged across trials for the same imagery images ($N = 20$). For the pooled results, to eliminate baseline and variability differences across subjects, the vividness scores obtained from individual subjects were first converted to z-scores within each subject, and then averaged across all trials from three subjects ($N = 60$). The rightmost two bars indicated as "artificial" and "natural" show mean vividness scores separately pooled for artificial shapes (15 artificial shapes) and natural images (10 natural images). Error bars indicate 95% confidence intervals across trials.
