# Supplementary material for: Deep image reconstruction from human brain activity
Source: PLoS Comput Biol. 2019 Jan 14;15(1):e1006633. doi: 10.1371/journal.pcbi.1006633 (PMC6347330; doi:10.1371/journal.pcbi.1006633)
Supplement: S18 Fig — Evaluations on individual subjects’ results are separately shown (VC activity; DNN 1–8; without the DGN; N = 15; chance level, 50%; cf., Fig 8D). Evaluations of reconstructions using pixel-wise spatial correlation showed 49.5%, 52.4%, and 53.8% for Subject 1–3, respectively. Evaluations of reconstructions using human judgment showed 85.6%, 84.4%, and 79.5% for Subject 1–3, respectively. (PDF) [file pcbi.1006633.s019.pdf]

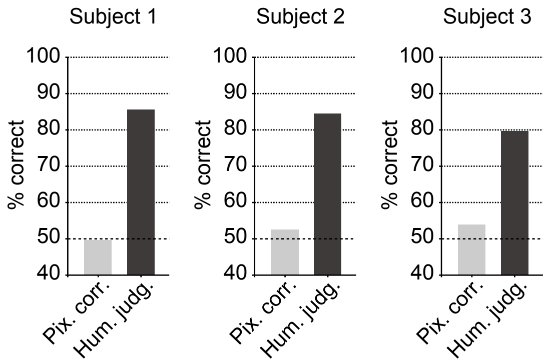

S18 Fig. Reconstruction quality of imagined artificial shapes for individual subjects. Evaluations on individual subjects' results are separately shown (VC activity; DNN 1–8; without the DGN; $N = 15$; chance level, 50%; cf., Fig 8D). Evaluations of reconstructions using pixel-wise spatial correlation showed 49.5%, 52.4%, and 53.8% for Subject 1–3, respectively. Evaluations of reconstructions using human judgment showed 85.6%, 84.4%, and 79.5% for Subject 1–3, respectively.
