# Supplementary material for: Deep image reconstruction from human brain activity
Source: PLoS Comput Biol. 2019 Jan 14;15(1):e1006633. doi: 10.1371/journal.pcbi.1006633 (PMC6347330; doi:10.1371/journal.pcbi.1006633)
Supplement: S19 Fig — Evaluations on individual subjects’ results are separately shown (VC activity; DNN 1–8; without the DGN; N = 15; chance level, 50%; cf., Fig 8E). Evaluations of reconstructions with respect to color showed 71.1%, 56.7%, and 66.7% for Subject 1–3, respectively. Evaluations of reconstructions with respect to shape showed 91.1%, 88.9%, and 81.1% for Subject 1–3, respectively. (PDF) [file pcbi.1006633.s020.pdf]

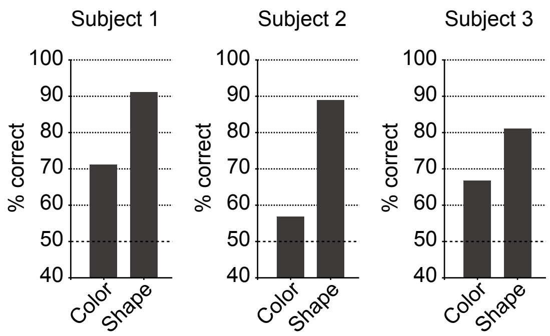

S19 Fig. Reconstruction quality of imagined artificial shapes for individual subjects separately evaluated for color and shape by human judgment. Evaluations on individual subjects' results are separately shown (VC activity; DNN 1–8; without the DGN; *N* = 15; chance level, 50%; cf., Fig 8E). Evaluations of reconstructions with respect to color showed 71.1%, 56.7%, and 66.7% for Subject 1–3, respectively. Evaluations of reconstructions with respect to shape showed 91.1%, 88.9%, and 81.1% for Subject 1–3, respectively.
