# Supplementary material for: Deep image reconstruction from human brain activity
Source: PLoS Comput Biol. 2019 Jan 14;15(1):e1006633. doi: 10.1371/journal.pcbi.1006633 (PMC6347330; doi:10.1371/journal.pcbi.1006633)
Supplement: S21 Fig — Evaluations on individual subjects’ results and their pooled result are separately shown (V1 activity; DNN 1–8; without the DGN; N = 15 for individual subjects and N = 45 for the pooled result; chance level, 50%; cf., Fig 8D). Evaluations of reconstructions using pixel-wise spatial correlation showed 48.2%, 51.3%, 48.4%, and 48.8% for Subject 1–3 and the pooled result, respectively. Evaluations of reconstructions using human judgment showed 57.7%, 73.5%, 60.1%, and 63.8% for Subject 1–3 and the pooled result, respectively. (PDF) [file pcbi.1006633.s022.pdf]

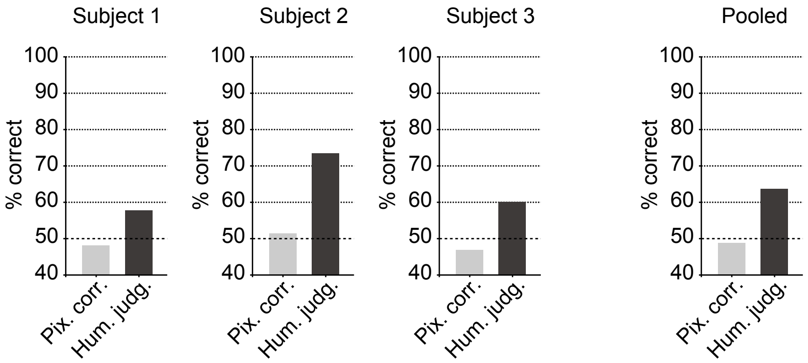

S21 Fig. Reconstruction quality of imagined artificial shapes (reconstructed from V1). Evaluations on individual subjects' results and their pooled result are separately shown (V1 activity; DNN 1–8; without the DGN; $N = 15$ for individual subjects and $N = 45$ for the pooled result; chance level, 50%; cf., Fig 8D). Evaluations of reconstructions using pixel-wise spatial correlation showed 48.2%, 51.3%, 48.4%, and 48.8% for Subject 1–3 and the pooled result, respectively. Evaluations of reconstructions using human judgment showed 57.7%, 73.5%, 60.1%, and 63.8% for Subject 1–3 and the pooled result, respectively.
