# Supplementary material for: Deep image reconstruction from human brain activity
Source: PLoS Comput Biol. 2019 Jan 14;15(1):e1006633. doi: 10.1371/journal.pcbi.1006633 (PMC6347330; doi:10.1371/journal.pcbi.1006633)
Supplement: S22 Fig — Evaluations on individual subjects’ results and their pooled result are separately shown (V1 activity; DNN 1–8; without the DGN; N = 15 for individual subjects and N = 45 for the pooled result; chance level, 50%; cf., Fig 8E). Evaluations of reconstructions with respect to color showed 60.0%, 56.7%, 55.6%, and 57.4% for Subject 1–3 and the pooled result, respectively. Evaluations of reconstructions with respect to shape showed 63.9%, 77.8%, 63.3%, and 68.3% for Subject 1–3 and the pooled result, respectively. As shown with the reconstructed images from VC (cf., Fig 8E), separate evaluations of color and shape reconstructions of artificial images from V1 also showed that shape rather than color had a major contribution to the high proportion of correct answers by human raters (three subjects pooled; two-sided signed-rank test, P < 0.05). (PDF) [file pcbi.1006633.s023.pdf]

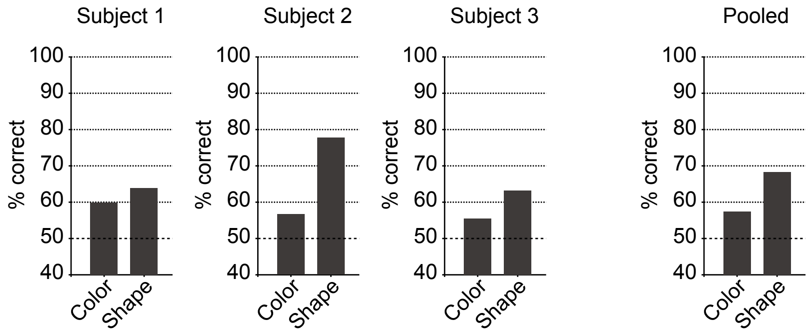

S22 Fig. Reconstruction quality of imagined artificial shapes separately evaluated for color and shape by human judgment (reconstructed from V1). Evaluations on individual subjects' results and their pooled result are separately shown (V1 activity; DNN 1–8; without the DGN; $N = 15$ for individual subjects and $N = 45$ for the pooled result; chance level, 50%; cf., Fig 8E). Evaluations of reconstructions with respect to color showed 60.0%, 56.7%, 55.6%, and 57.4% for Subject 1–3 and the pooled result, respectively. Evaluations of reconstructions with respect to shape showed 63.9%, 77.8%, 63.3%, and 68.3% for Subject 1–3 and the pooled result, respectively. As shown with the reconstructed images from VC (cf., Fig 8E), separate evaluations of color and shape reconstructions of artificial images from V1 also showed that shape rather than color had a major contribution to the high proportion of correct answers by human raters (three subjects pooled; two-sided signed-rank test, $P < 0.05$).
