# Supplementary material for: Deep image reconstruction from human brain activity
Source: PLoS Comput Biol. 2019 Jan 14;15(1):e1006633. doi: 10.1371/journal.pcbi.1006633 (PMC6347330; doi:10.1371/journal.pcbi.1006633)
Supplement: S1 Fig — DNN feature decoding accuracy obtained from VC activity was evaluated by the correlation coefficient between the true and decoded feature values of each feature unit following the procedure in Horikawa & Kamitani (2017) [10]. The evaluation was individually performed for each of the three types of seen images (natural images, artificial shapes, and alphabetical letters) and each of the two types imagery images (natural images and artificial shapes). Correlation coefficients were averaged across units in each DNN layer. The mean correlation coefficients are shown for each types of layers (error bars, 95% confidence interval [C.I.] across units; N of each layer equals to the number of units in each layer; see Material and Methods: “Deep neural network features” for details). (PDF) [file pcbi.1006633.s002.pdf]

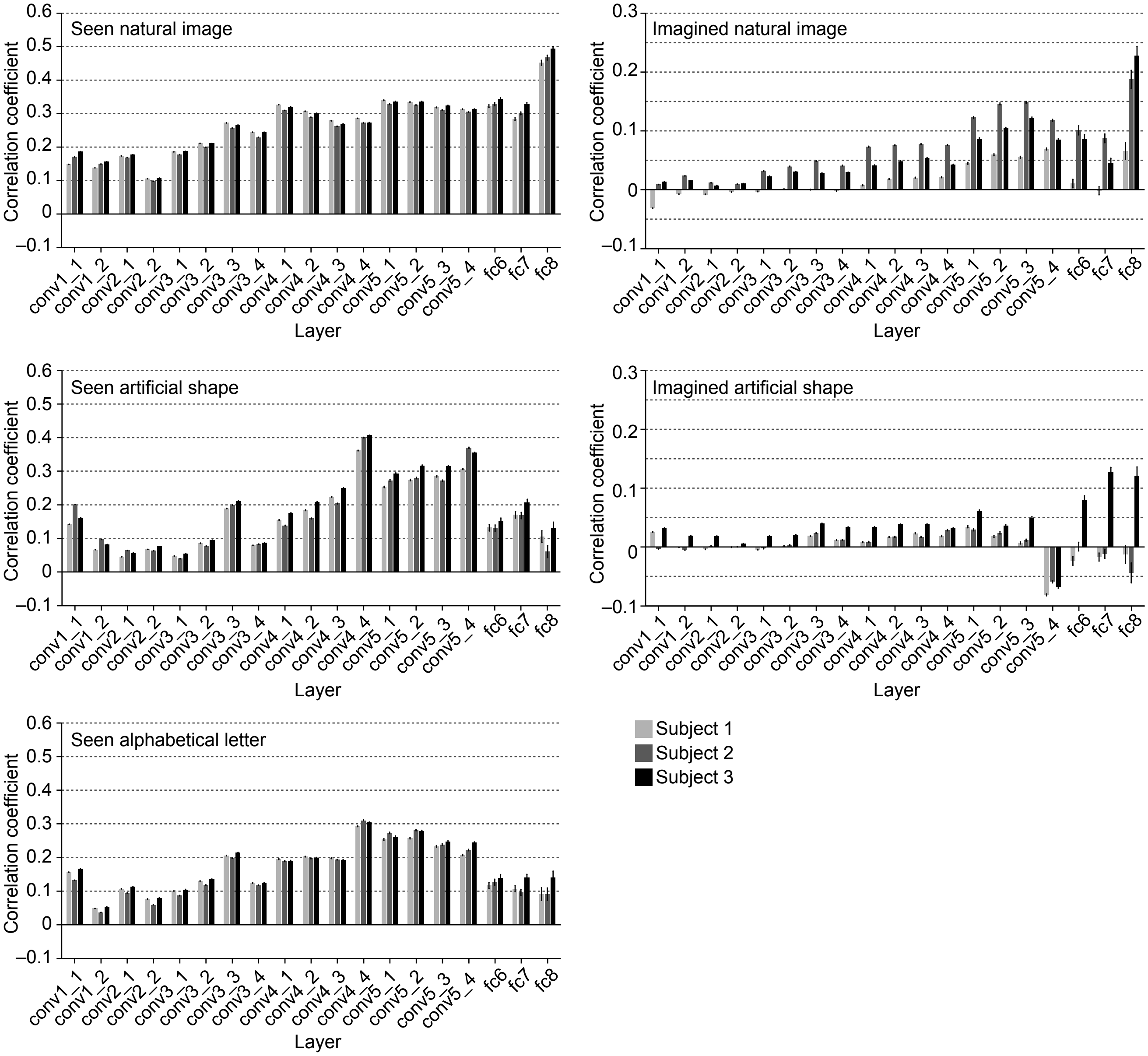

S1 Fig. DNN feature decoding accuracy. DNN feature decoding accuracy obtained from VC activity was evaluated by the correlation coefficient between the true and decoded feature values of each feature unit following the procedure in Horikawa & Kamitani (2017) [12]. The evaluation was individually performed for each of the three types of seen images (natural images, artificial shapes, and alphabetical letters) and each of the two types imagery images (natural images and artificial shapes). Correlation coefficients were averaged across units in each DNN layer. The mean correlation coefficients are shown for each types of layers (error bars, 95% confidence interval [C.I.] across units; *N* of each layer equals to the number of units in each layer; see Material and Methods: “Deep neural network features” for details).
